# High level protein-purification allows the unambiguous polypeptide determination of latent isoform PPO4 of mushroom tyrosinase^☆^

**DOI:** 10.1016/j.phytochem.2013.12.016

**Published:** 2014-03

**Authors:** Stephan G. Mauracher, Christian Molitor, Claudia Michael, Martin Kragl, Andreas Rizzi, Annette Rompel

**Affiliations:** a Department of Biophysical Chemistry, University of Vienna, Althanstraße 14, 1090 Vienna, Austria; b Department of Analytical Chemistry, University of Vienna, Währinger Straße 38, 1090 Vienna, Austria

**Keywords:** PPO, polyphenol oxidase, *ab*, *Agaricus bisporus*, A-TYR, active tyrosinase, L-TYR, latent tyrosinase, Metalloenzyme, *Agaricus bisporus*, Latent tyrosinase, Tyrosinase maturation, Zymogen, PPO4, Type-3 copper center, Post translational modification, Polyphenols, Protease inhibitor

## Abstract

Tyrosinases catalyze two initial reaction steps in the formation of melanin. Purification of tyrosinases had always been a process accompanied with various problems caused by enzymatic browning processes. Here, an approach is presented for the purification of the latent enzyme from mushrooms which averts and removes interfering compounds (e.g. polyphenols) in advance to the extraction process. The described method is supposed being well suitable as a general protein purification protocol from natural sources like fungi and plants.

The purified enzyme was investigated in detail by means of mass spectrometry: its intact protein mass was determined as 64,247.3 Da and it was identified as number four of in total six isoforms (PPO1–6) by means of sequence analysis. Some PTMs, strain specific sequence disparities and several cleavage sites including the one causing enzyme-activation (Ser^383^) were determined, thus, providing insights on the maturation process of this latent tyrosinase zymogen. Based on these sequence data it can be concluded that the polypeptide backbone of the latent form of the tyrosinase PPO4 ranges from Ser^2^ to Thr^565^, missing when compared to the gene-derived sequence a small part (46 amino acids) of the *C*-terminal tail. The high content on hydrophobic amino acids within this missing tail gives rise to speculations whether this part might have a function as a membrane anchor.

## Highlights

• An innovative method for protein purification from natural sources.
• Indications that full length PPO4 has *in vivo* a membrane orientated origin.
• Mass spectrometric measurements provide exact cleavage sites for maturation.
• Revealing PTMs in the protein backbone and strain specific sequence heterogeneity.

## Graphical abstract

Mushroom tyrosinase diversity – six precursor isoforms, all capable of being activated and generating protein purification interfering compounds – is taking one step further to reconnaissance. This innovative method for protein purification provides access to latent tyrosinase from natural sources and enabled experiments revealing insights in a hitherto uncharacterized isoform.

**Figure:**
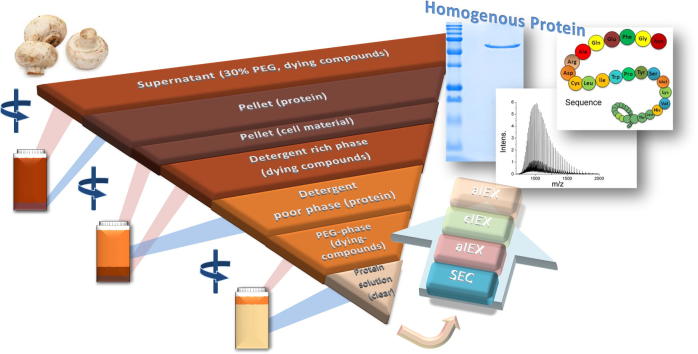

## Introduction

Tyrosinase (EC 1.14.18.1; EC 1.10.3.1) is an enzyme which is distributed over a wide range of organisms from bacteria, fungi, and plants to mammals (Mayer, 2006; Oetting, 2000). It catalyzes the first reaction steps in the formation of the polyphenolic pigment compounds known as melanins (Sánchez-Ferrer et al., 1995; Seo et al., 2003). These pigments are formed during senescence of agricultural products lowering significantly their disposal value. To oppose this matter a lot of effort has been put into elucidating the functional and structural properties of this enzyme. Numerous attempts have been performed to find appropriate inhibitors against the enzyme’s activity in respect on functionality, toxicity, availability and expense, however with minor success (Chang, 2009; Khan, 2007; Kim and Uyama, 2005; Schurink et al., 2007).

**Scheme 1:**
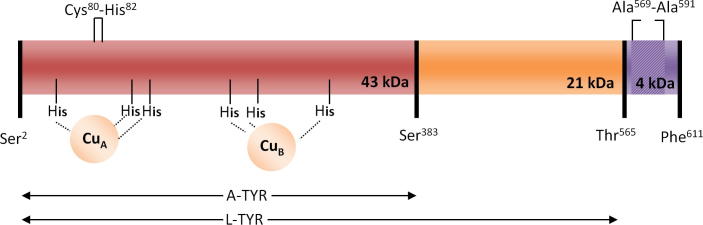
Schematic illustration of the polypeptide chain of PPO4 mushroom tyrosinase. The polypeptide chain of active tyrosinase (core-region) is colored in red. It ranges from Ser^2^ to the determined cleavage site at Ser^383^. This A-TYR part contains both copper binding sites forming the active site as well as the thioether bridge between Cys^80^ to His^82^. The *C*-terminal part is colored in orange. It starts with position Glu^384^ and ends with the cleavage site at Thr^565^. The untraceable *C*-terminal tail above Thr^565^ is colored purple. Amino acids of this part were not found during this investigation. Computer based structure modeling of this sequence tail suggests the presence of a trans-membrane helix between Ala^569^ and Ala^591^. (For interpretation of the references to color in this figure legend, the reader is referred to the web version of this article.)

Currently the amino acid sequences of six polypeptides (PPO1 to 6) are known for a polyphenol oxidase originating from *Agaricus bisporus* (Genome Sequence, n.d.; Weijn et al., 2013; Wichers et al., 2003; Wu et al., 2010). It is known that the protein exists as a zymogen, also described as the latent form (L-TYR, named *pro*-tyrosinase by others (Fujieda et al., 2013)), and an active form (A-TYR) (for a schematic illustration see Scheme 1) (Bouchilloux et al., 1963; Mallette and Dawson, 1949; Yamaguchi et al., 1970). The mature active form is generated by proteolytic cleavage of the latent form, by which the *C*-terminal part of the protein is removed and the previously buried active site becomes exposed (Faccio et al., 2013; Fujieda et al., 2012). An artificial activation can be achieved due to the use of detergents (Espín and Wichers, 1999; Moore and Flurkey, 1990). Recently, homology models of PPO1, 2, 4, 5 were published (Inlow, 2012) which in all cases predict an orientation of the *C*-terminal chain in a way shielding the active site. Thus, for the purpose of developing a commercially relevant technology for suppressing the enzymatic browning reactions, the putative more promising way could be a strategy of hindering the proteolytic activation process itself rather than inhibiting the activity of the active form. For this purpose the knowledge of the crystal structure of the latent tyrosinase would be beneficial as it was shown before for *pro*-tyrosinase from *Aspergillus oryzae* (Fujieda et al., 2013) and a closely related type-3 copper protein from *Manduca sexta* (Li et al., 2009).

Recently, the first crystal structure of an active eukaryotic tyrosinase (*A. bisporus*) was published (Ismaya et al., 2011). The protein was found being present in a complex as described earlier (Strothkamp et al., 1976) consisting of two heavy (H) and two light subunits (L) forming a tetramer of the type (H_2_L_2_). The heavy subunit containing the active site corresponds to the amino acids 2 to 392 from the polyphenol oxidase 3 (PPO3) sequence which covers *in toto* 576 residues (Wu et al., 2010). The investigated enzyme was found being in the deoxy state (Solomon et al., 1996). Interestingly, the heavy subunit shows “stand alone” activity. No evidence was found for the presence of the *C*-terminal part (residues 393–576) of the protein. The small subunit, L, corresponds to ORF239342 found in the 2010 generated genome sequence of *A. bisporus* (Genome Sequence, n.d.). Neither its cell based origin (or whether it’s an artificial protracted protein during the purification process), nor the function of this lectin like fold small protein could be clarified by the authors (Ismaya et al., 2011).

Isolation and purification of mushroom tyrosinase is a process known over long time and well developed, however, only up to a certain point of purity (Espín and Wichers, 1999; Fan and Flurkey, 2004; Haghbeen et al., 2004; Nelson and Mason, 1970). It is readily feasible to get highly active protein extracts, however, these extracts are heterogeneous in composition (with respect to protein isoforms and particularly impurities), are instable and their activity determination is hardly reproducible (Flurkey et al., 2008; Flurkey and Inlow, 2008; Rescigno et al., 2007). Such tyrosinase extracts are also commercially available (*Sigma–Aldrich*). Preventing sample heterogeneity when preparing these extraction products is a challenging task because of several reasons outlined below.

First, at the genetic level, six different isoforms exist of the enzyme, i.e. PPO1 to 6 (Weijn et al., 2013; Wichers et al., 2003; Wu et al., 2010). Most likely, all of them are proteolytically processed, which results in differing active and latent forms. Expression studies have shown (Li et al., 2011; Weijn et al., 2013) that these different isoforms (PPO1–6) are expressed in different quantities depending on the growth-stage and the fruit-body compartment (e.g. cap, stipe) (Hammond and Nichols, 1976). Second, the formation of the active form of the protein by proteolytic cleavage starts immediately after cell lysis forming great numbers of active tyrosinase species as well as huge amounts of colored compounds (e.g. melanins). This process is hardly preventable. In addition, the accruing heterogeneity of the protein might be due to further unspecific cleavages, post translational modifications and protein–protein aggregation. Third, tyrosinase is known for protein cross-linking as well as for its potential of oxidizing protein backbone tyrosines (Gieseg et al., 1993; Jus et al., 2011; Thalmann and Lötzbeyer, 2002). Recently, cross-links in recombinant tyrosinase of *A. oryzae* has been confirmed by means of mass spectrometry (MS) (Fujieda et al., 2012). Protein aggregation and backbone tyrosine oxidation conceivably are reasons for the observed post-browning effect which occurs reproducibly after removing reducing agents by e.g. size exclusion chromatography (SEC) and temporal storage of the protein in solution (Sojo et al., 1998). Another major reason for inhomogeneity, instability and irreproducibility of tyrosinase extracts lies in the difficulty to remove the brown colored compounds generated by oxygen exposure after cell disruption (Espín and Wichers, 1999; Wichers et al., 1996; Zhang and Flurkey, 1999). Such pigments are formed by polymerization of quinones. These quinones and their polymerized products are known to form protein–protein aggregates, cause protein precipitation, simply bind to proteins and also color and disable chromatographic materials irreversibly (Fujieda et al., 2012; McManus et al., 1981). Hence, it can be argued that the occurring browning reactions are not purposed to retain cell functionality than rather stifle all functional processes including pathogenic ones (Bell and Wheeler, 1986). Therefore, it is reasonable to oppose tyrosinase activation and subsequent browning processes in order to isolate and purify proteins in their physiological homogenous forms.

In this work a new and significantly improved approach for isolating and purifying latent mushroom tyrosinase will be presented which focuses on averting the formation of interfering pigments and their subsequent products and/or on removing them. Moreover, the presented method achieves protein homogeneity by separating occurring isoforms. The extraction approach is partially based on a method published elsewhere but is modified and optimized in several respects (Sojo et al., 1998). The method described in this paper allows a very efficient separation of L-TYR from A-TYR. After purification to proximate homogeneity the protein could be identified and characterized by MS based methodology providing sequence information, precise cleavage positions and the accurate mass of the intact protein (A-TYR and L-TYR) with its fringed *C*-terminus expounding another conclusion for protein heterogeneity. Several modifications as well as severe sequence disparities (point-mutations) due to differing strain origin could be determined by means of MS data. In addition, some predictions regarding secondary structures and membrane embedding which are based on computational model calculations will be added.

## Results

### Initial sample preparation, extraction and processing

The pre-extraction method was aimed at preparing a lyophilized mushroom powder. An efficient cell disruption (effected by using high amounts of triton-X114 (8%) and by repetitive mixing steps) led to high amounts of protein accessible for the subsequent extraction. The pre-extraction including a treatment with a highly concentrated PEG solution (proteins not soluble) led to an intensely colored supernatant containing colored compounds and putative natural tyrosinase substrates. The triton-X114 phase separation together with the repetitive PEG phase separations were able to brighten up the protein solution step by step by decreasing the pigment content of the sample. The chosen pH-value of 5.3 of the extraction buffer supported this de-coloring process and prevented also post browning after removal of sodium ascorbate by SEC.

### Chromatographic purification (FPLC)

**Fig. 1:**
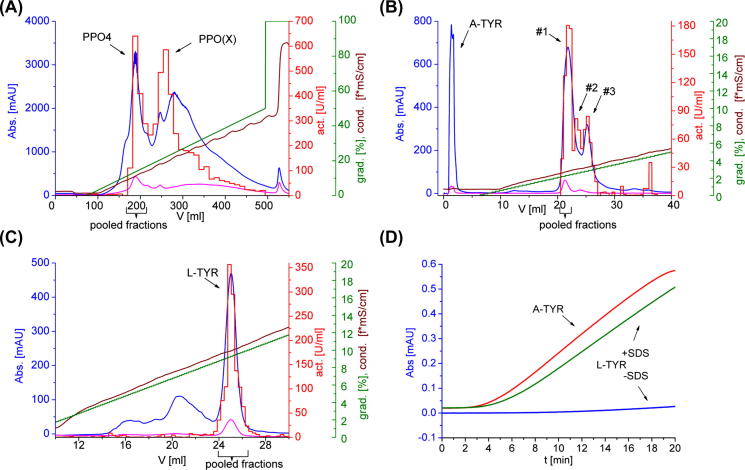
Chromatographic separation steps and enzymatic assay. (A) AEX chromatography using Q-Sepharose stationary phase. (B) CEX chromatography using MonoS stationary phase. (C) AEX chromatography using MiniQ stationary phase. Legend:  UV absorbance at 280 nm [mAU],  UV absorbance at 345 nm [mAU],  activity [U/mL],  gradient [% buffer B],  conductivity [mS/cm^2^] (*f* = ∼0.5 * grad.). (D) Enzymatic assay of the final purified protein samples (A-TYR, L-TYR) with l-tyrosine as substrate. (Absorbance vs. time at 305 nm).  A-TYR (50 mM potassium phosphate buffer pH 6.5),  L-TYR (50 mM potassium phosphate buffer pH 6.5),  L-TYR (10 mM sodium phosphate buffer + 2 mM SDS, pH 6.5).

The entire chromatographic purification procedure consisted of four subsequent steps. For the first step a SEC column was directly connected to an anion exchange (AEX) column filled with DEAE–Sepharose. The applied SEC method can be considered as the final step for removing interfering low molecular weight compounds including the remaining ammonium sulfate. The AEX run was operated by a 3-step elution program with increasing ionic strength established a fast and rough removal of non-target proteins including proteases (chromatogram not shown). The target-protein eluted in the middle step with 0.2 M sodium chloride. The direct connection of SEC with AEX enhanced the recovery of the latent form, L-TYR. In the subsequent AEX run with Q-Sepharose as stationary phase, two major fractions exhibiting tyrosinase activity were monitored (Fig. 1A). Activity measurements of the pooled fractions belonging to the earlier eluted peak (indicated with “PPO4” in Fig. 1A) showed a high grade of latency (93%). These fractions were used for further purification. The pooled fractions of the second peak (indicated with “PPO(X)” in Fig. 1A) showed rather low latency (12%) and probably contained other active isoforms (e.g. PPO3); they were discarded. Peak one was subjected to a subsequent high-resolution cation exchange (CEX) purification step, using MonoS as stationary phase. This procedure led to the separation of three different forms of L-TYR (#1, #2, #3 in Fig. 1B), while A-TYR was found in the pass through fractions (lower p*I*, see Table 19c in Supporting information). The first of these peaks, #1, was collected and further purified by a final chromatographic step consisting of high resolution AEX chromatography using a MiniQ stationary phase (Fig. 1C). After this step the protein was used for the subsequent structural characterization experiments.

Applying this extraction and purification method to 300 g (standard batch) of lyophilized mushrooms (stipes) yields approximately 2 mg of L-TYR isoform PPO4. Notably, also A-TYR (1–2 mg) and other species of the enzyme (e. g. PPO(X) and #2, #3 in Fig. 1A/B) are co-purified.

### Enzymatic activity assay

The enzymatic monophenolase activity of this purified tyrosinase form (L-TYR) towards l-tyrosine as substrate showed a latency grade of 93% (Fig. 1D) (using for the determination of the latency a comparison between SDS and non-SDS containing environments).

### Gel electrophoresis

**Fig. 2:**
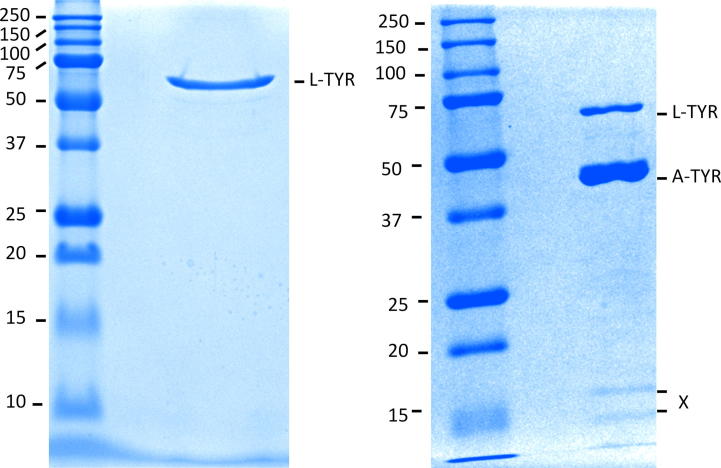
Analytic SDS–PAGE. (A) Purified L-TYR (62 kDa) after MiniQ run. (B) A-TYR (44 kDa) after passing through MonoS column (still containing L-TYR). X → low molecular weight (∼15 kDa) bands (ABL, PPO4 fragments). Staining: Coomassie Brilliant Blue. *M*_w_ marker [kDa] in the respective left lanes.

SDS–PAGE analysis of the finally purified L-TYR revealed the presence of one single band only (Fig. 2A) with a molecular mass of approximately 62 kDa. However, A-TYR in comparison showed still little contamination with some L-TYR species and a mass of about 44 kDa (Fig. 2B) as well as some faint bands at low molecular masses.

### Protein identification and sequence confirmation

**Fig. 3:**
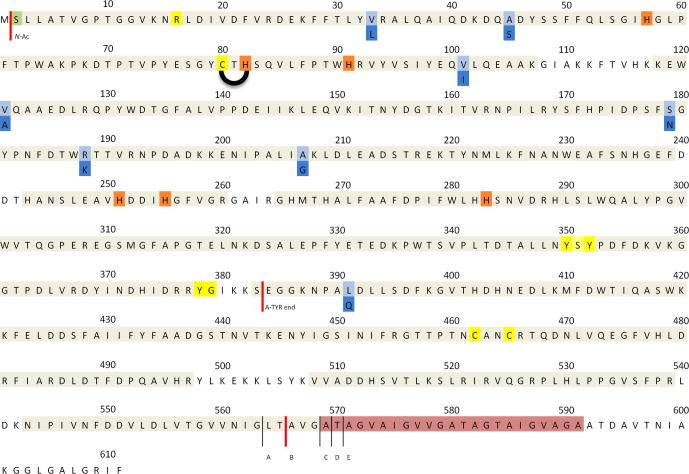
Sequence of PPO4. Highlighted are:  – tyrosinase common motifs;  – copper coordinating histidines;  – acetylated *N*-terminus; / – Mutations (PPO4/K9I869);  – the putative trans-membrane helix;  – Peptides identified by HPLC–ESI-MS/MS are highlighted gray. Red thick lines () indicates the start/end of the L-TYR and A-TYR sequence, respectively, as deduced from matching with the molecular mass determined for the isolated protein by ESI-QTOF. Black thin lines () indicate the cleavage positions of the minor sub-species (A, C–E) of L-TYR. (For interpretation of the references to color in this figure legend, the reader is referred to the web version of this article.)

HPLC–ESI-MS/MS experiments carried out with a LTQ-Orbitrap, clearly identified the isolated and purified protein as PPO4 mushroom tyrosinase (UniProt.: C7FF05, GenBank: ACU29458.1) yielding a score of 468 (−10lgP) and a maximum sequence coverage of 95%. 75 tryptic peptides were identified by accurate matching of the molecular mass (the large majority within 2.5 ppm) and their MS/MS pattern. They are highlighted gray in Fig. 3 (the comprising list is shown in Table 1). Besides almost complete peptide coverage of the main core region (Ser^2^–Ser^383^), also 33 partial overlapping peptides in the *C*-terminal part, defining the enzymes latent form, were found. No tryptic peptides were detected and identified beyond Ala^574^ (Table 1 and Fig. 3). At the *C*-terminal end of the protein nearly all possible non-tryptic peptides were found, i.e., from Asn^543^–Ala^574^, suggesting a highly proteolytically fringed *C*-terminus. This result was also confirmed by the accurate mass determination of the intact protein described below. Additionally, several modifications were concluded from the tryptic peptide spectra. All modifications are marked in Fig. 3. The *N*-terminal starting peptide (Ser^2^–Lys^14^) lacking the Met^1^ showed a very high probability (−10lgP = 72.2) of being acetylated as was reported for TYR from *Neurospora crassa* (Lerch et al., 1982). Peptide Asp^69^–Arg^92^ clearly contained the common thioether bridge (Cys^80^–His^82^) found for all known eukaryotic PPOs according to literature (Gielens et al., 1997; Ismaya et al., 2011). Interestingly, a mutational isoform seems to be co-existently present in which eight amino acids are exchanged (see below). Both, mutated as well as the non-mutated peptides were identified with high scores (−10lgP) (Table 1). By blasting the mutated PPO4 sequence a perfect match with the so far uncharacterized protein K9I869 (UniProt.) derived from the *A. bisporus* genome sequence of strain H97 could be established (Morin et al., 2012). The only two cysteines able to form a disulfide bridge (Cys^462^–Cys^465^) were actually found being present with the “closed” disulfide-bridge, i.e. exhibiting a molecular mass of 2.02 Da less than the mass calculated for the “open bridge” variant. Though in this instance the measured peptide masses gave a deviation from the calculated values of 25 ppm, the fragment spectrum exhibited several b and y ions matching with the predicted *m*/*z* ratios and provided in this way evidence for peptide identity.

In addition to the identification of the target protein band (L-TYR) also the faint bands in the A-TYR lane were identified to clarify if a putative attachment of the small subunit (UniProt: G1K3P4, Ismaya et al., 2011)) might happen after the removal of the *C*-terminus. Peptides of PPO4 (main core and *C*-terminal) as well as of the small (16.2 kDa) protein *ab*Lectin (ABL, UniProt: Q00022, sequence coverage: 73%) were found. No peptides corresponding to the small subunit (Q00022) were detected.

The comprehensive set of protein identification data containing lists of all found peptides is presented in the Supporting information.

### Molecular mass of intact L-TYR

**Fig. 4:**
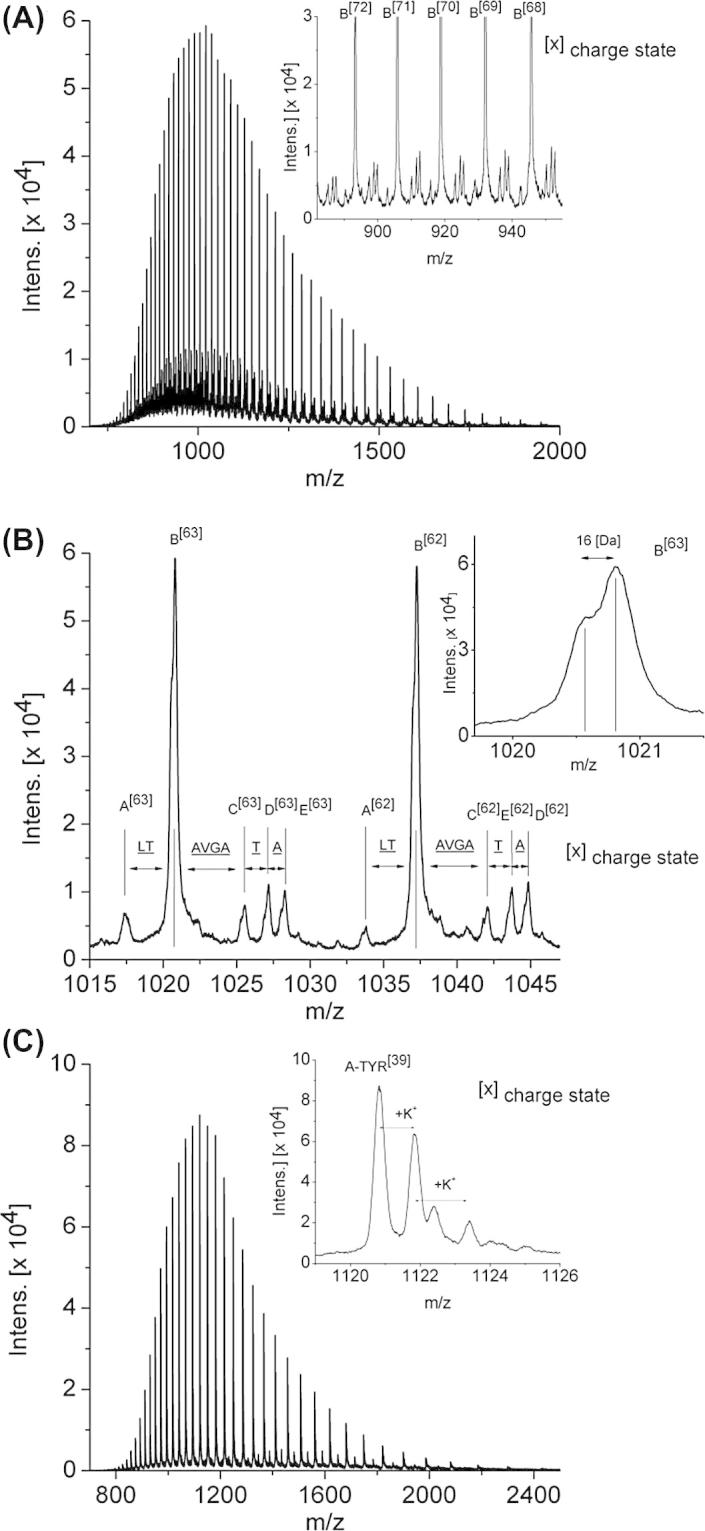
ESI-QTOF mass spectra of intact L-TYR and A-TYR. (A) Entire mass spectrum of L-TYR (PPO4). Panel (a): Peaks of charge states [72] to [68] magnified. (B) Zoomed section of charge states [63] and [62]. Masses of species A–E: A = 64,034 Da, B = 64,247.3 Da; C = 64,546 Da; D = 64,647 Da; E = 64,718 Da. Mass differences between species fit to distinct amino acid composition indicated in amino acid letter code. Panel (b): Highly zoomed figure of charge state [63] showing clearly a shoulder corresponding to mass difference of PPO4 and K9I869. (C) Entire mass spectrum of A-TYR. Panel (c): Magnified charge state [39] of A-TYR displaying several potassium adducts (mass differences ∼39 Da).

The mass spectrum of the entire protein present in the finally purified L-TYR and A-TYR fractions is given in Fig. 4. It was gained by the described ESI-QTOF instrument with resolution (FWHM) of 40,000 in this mass range and a mass accuracy of better than 5 ppm. The charge state distributions depicted in panel (a) of Fig. 4A (ranging from about 50 to up to more than 80 charges) indicate the presence of one major protein species (B, when assorting by increasing M_r_) and four minor abundant ones (A, C–E) with signal intensity ratios of about 6:1. A zoomed-in section of this spectrum is shown in Fig. 4B. The magnified panel (b) in Fig. 4B shows unambiguously that each species peak has a shoulder caused by a preceding peak (Δ*m* ∼16 Da). For the deconvolution of the charge state distribution shown in panel (a) of Fig. 4A, 28 distinct peaks were used for the major species B, and at least 17 peaks with sufficient signal to noise ratios for the species A, C–E. Assuming that these positive charge (z) states are solely caused by the attachment of z protons the average molecular masses for the major species B can be assessed as 64,247.3 Da (STD less than 0.2 Da based on 5 measurements) and for A = 64,034 Da; C = 64,546 Da; D = 64,647 Da; E = 64,718 Da (STD less than 1.5 Da), respectively. Because of the acidic electrospray conditions (0.05% formic acid) it is likely that the originally present Cu^2+^ ions are not longer present in the analyte ions in gas phase. This could be confirmed by non-acidic sample treatment resulting in a determined mass of approximately 160 Da (2x Cu^2+^, 1x ${\text{O}}_{2}^{2-}$) higher. However, the ionization yield under non-acidic conditions was low. Thus, the thereby gained data possessed a low signal to noise ratio and were insufficient for accurate mass determination of the Cu containing protein (see Supporting information).

Calculation of the theoretical average mass of the L-TYR PPO4 isoform consisting of (i) the polypeptide backbone ranging from Ser^2^ to Thr^565^ (taken the sequence confirmed by our sequence analysis discussed above), (ii) the presence of a thioether bridge between Cys^80^ and His^82^ (reported in the literature for many type-3 copper proteins i.e. *ab*PPO3, molluscan hemocyanin and confirmed here) leading to a loss of two H atoms (−2.02 Da) as well as a disulfide bridge (−2.02 Da), (iii) the presence of an acetylated *N*-terminus (+42.01 Da), results in a value of 64,233.2 Da (Gielens et al., 1997; Ismaya et al., 2011; Klabunde et al., 1998; Wu et al., 2010).

Taking into account the presence of the eight sequence disparities of K9I869 to PPO4 (Table 1) (i.e., Val^33^ → Leu (Δ*m* = 14.02 Da), Ala^45^ → Ser (Δ*m* = 15.99 Da), Val^101^ → Ile (Δ*m* = 14.02 Da), Val^121^ → Ala (Δ*m* = −28.03 Da), Ser^179^ → Asn(Δ*m* = 27.01 Da), Arg^188^ → Lys (Δ*m* = −28.01 Da), Ala^207^ → Gly (Δ*m* = −14.02 Da) and Leu^391^ → Gln (Δ*m* = 14.97 Da)), a total difference between those two isoforms of 15.95 Da is given. The calculated value for the intact mutated PPO4 (K9I869) protein would therefore be 64,249.2 Da. The difference in mass of the mutated (K9I869) and the non-mutated (PPO4) form matches perfectly to the found shoulder shown in panel (b) of Fig. 4 B (Δ*m* ∼16 Da).

Comparing the measured mass for species B with the mass calculated for the K9I869 polypeptide under the mentioned conditions, a difference of less than 1.9 Da is found. This difference is probably owing to a slight shift of the peak maxima due to the incomplete resolution of the shoulder.

The minor sub-species (A, C–E) are reasonably deduced as protein forms being proteolytically cleaved at different positions of the *C*-terminal polypeptide chain as specified in Fig. 4B. The mass differences between these proteolytic species fit accurately to the amino acids indicated.

ESI mass spectra for A-TYR are shown in Fig. 4C. The estimated mass for A-TYR is 43,673.1 Da (STD less than 0.5 Da) fitting to the polypeptide backbone Ser^2^–Ser^383^ again accounting a thioether bridge and an acetylated *N*-terminus with deviation of 0.2 Da (PPO4) and −0.8 Da (K9I869), respectively. Other detectable peaks shown in panel (c) of Fig. 4C correspond to potassium adducts.

### Computational structure modeling

From the molecular mass of the isolated “intact” protein as well as from the sequence analysis it becomes very likely that the major species’ (B) final *C*-terminal amino acid is Thr^565^. The sequence of the following amino acids of PPO4 towards the *C*-terminus (i.e. Thr^565^–Phe^611^) – this part of the protein is widely missing according to the two MS experiments – indicates that this *C*-terminal part of the protein mainly consists of a continuous sequence of hydrophobic amino acids (Wu et al., 2010).

**Fig. 5:**
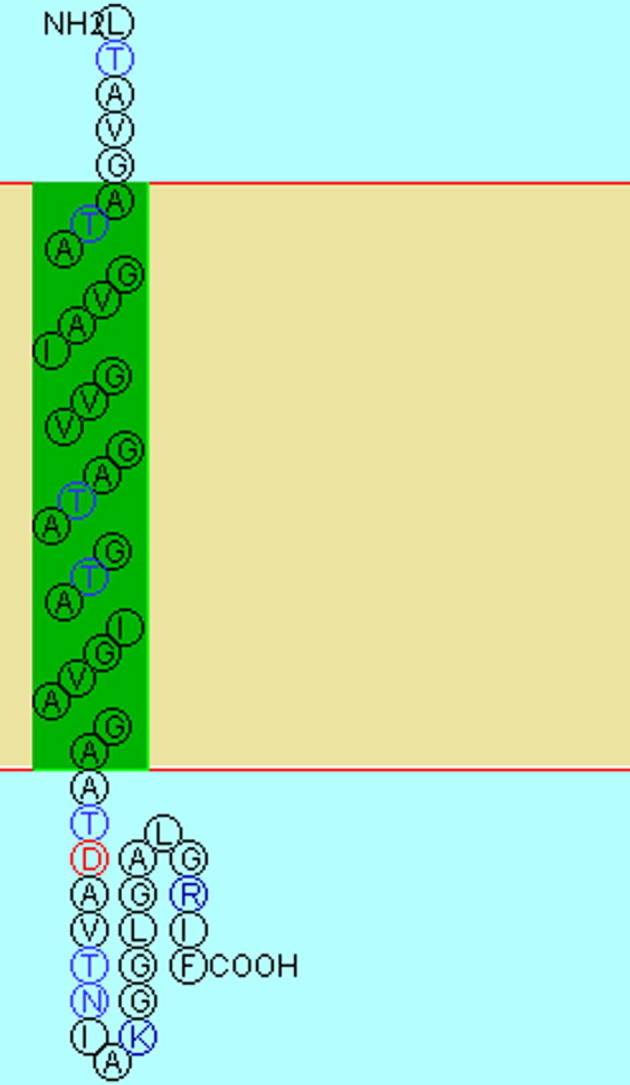
Trans membrane anchor of L-TYR (PPO4). Sequence in the *C*-terminal tail of PPO4 (Ala^569^ to Ala^591^). For this range a trans-membrane helix structure is predicted by model calculations (green highlighted area). (For interpretation of the references to color in this figure legend, the reader is referred to the web version of this article.)

The pronounced hydrophobicity of this missing sequence Thr^565^–Phe^611^ is illustrated by a calculated grand average hydropathicity index (GRAVY) of 1.128. For the purpose of comparison, the GRAVY indices for the protein L-TYR as isolated here (i.e. Ser^2^–Thr^565^) is −0.438 and that of the activated form A-TYR (covering Ser^2^–Ser^383^) is −0.505. Interestingly, the results of a computational structure prediction program propose that the continuous hydrophobic part of this missing sequence, namely Ala^569^ to Ala^591^, form an integral membrane helix as illustrated in Fig. 5.

## Discussion

A new and improved method for the isolation of latent tyrosinase (L-TYR, PPO4) from white edible mushrooms is presented here. Particular emphasis was on establishing a method for the consequent and effective removal of pigments and dying compounds prior to the chromatographic protein purification steps. This very initial preparation step of the mushroom powder is crucial. By the removal of present pigments and their potential precursor-compounds (e.g. substrates like l-tyrosine or tyramine) interfering and troubling reactions can be prevented in advance. This removal could be achieved by introducing a compound-extraction step with highly concentrated PEG solutions (proteins not soluble) and lowering the pH values of the extraction buffers from the commonly used values of 6.5–7.0 to 5.3 (Haghbeen et al., 2004). The protein-extraction following repetitive detergent- and aqueous polymer-phase separations were decisive for getting rid of these colored substances (pigments). In this way, the usually occurring browning reactions could be significantly decreased compared to the established methods for tyrosinase extraction (Espín and Wichers, 1999; Fan and Flurkey, 2004; Haghbeen et al., 2004; Nelson and Mason, 1970). The applied method led to clear protein solutions with low monophenolase activity which is, however, drastically increased by the presence of SDS in the enzymatic assay solution (pH 6.5, 2 mM SDS). This result indicates a high content of L-TYR and low content of A-TYR. Moreover, the post-browning effect, which otherwise occurs routinely after the removing of reducing agent (e.g. sodium ascorbate) and temporal storage, could be prevented by the described method (Fujieda et al., 2012; Gieseg et al., 1993; Sojo et al., 1998). The lower pH of 5.3 was helpful in slowing down these browning reactions since the reported optimum of PPO activity lies at pH values between 6.3 and 7.5. Stability of the enzyme is still given at this pH whereas autooxidation of diphenols is significantly slower at lower pH values (Jeon et al., 2005). A post-browning reaction taking place after the SEC due to the change in pH (5.3–7.5) could be prevented, most likely by the rapid purification. This was facilitated by directly connecting the SEC with the first AEX (DEAE–Sepharose), by the fast elution protocol, and by the subsequent second AEX (Q-Sepharose) step effecting the actual isoform (PPO4 from PPO(X), e.g. X = 3) separation (Fig. 1A). In this way no long temporary storage gaps are needed and the enzyme can widely be kept in its latent state. The presumption that this post-browning effect is due to the oxidation of tyrosine residues (by the enzyme itself) to quinones, can be supported. This is due to the mere fact that the described extraction and purification method suppresses these processes. Consequently no browning could be visually observed.

In the course of the chromatographic separations there were several fractions exhibiting SDS-induced monophenolase activity. The presence of different enzyme species of L-TYR (e.g., partially cleaved forms) has to be expected. This is illustrated by the high resolution CEX chromatogram shown in Fig. 1B (A-TYR passed through, fractions containing #2 and #3 were not characterized). Even after the final purification and polishing step (MiniQ, Fig. 1C) several sub-species (cleaved forms) are still present as shown by the MS data in Fig. 4A, B and Table 1. Are there other potential sources for this remaining diversity?

(i) It is not likely that these forms result from protein cross-linking or from protein–pigment interactions, or amino acid backbone oxidation, as the proposed method established all possible effort to hinder these processes and mass spectrometric data does not indicate any supporting evidence for that. Partial methionine oxidation found in the tryptic maps (supp. dat.) of L-TYR might occur during sample preparation as analytical artifacts.
(ii) The observation that the presence (and amount) of differing protein forms was not significantly reduced upon generously increasing the amount of protease inhibitors. This fact indicates that the proteolytic *in vitro* cleavage processes occurring during the extraction and purification steps were not the major source of protein heterogeneity (Espín and Wichers, 1999; Jolley et al., 1969; Van Leeuwen and Wichers, 1999; Wichers et al., 1996; Zhang and Flurkey, 1999).
(iii) It is likely that several activated isoforms (predominantly PPO3 and 4) were already present in the fungi.

However, the heterogeneity observed in the finally purified PPO4 sample (five sub-species distinguished by MS) is clearly caused by proteolytic cleavage at the *C*-terminal side. This processing happens most likely *in vivo* in pre- or post-harvesting states (Hammond and Nichols, 1976; Heneghan et al., 2008; Kingsnorth et al., 2001). *In toto*, we do not assume that the final length of the polypeptide chain in our final purified protein (or any other modification despite methionine oxidations) was induced by the analytical purification protocol.

Gel electrophoresis resulted in one single band at 62 kDa, showing that the protein sample was properly liberated of any non-target protein of different size (Fig. 2A). When considering the theoretical masses of L-TYR (∼68.3 kDa) and A-TYR (∼43.5 kDa), the gel electropherogram of L-TYR indicates that the protein is not proteolytically activated to a significant extent. A sample of the non-bound (MonoS run) pooled fractions exhibited a strong colored band (44 kDa) and weak colored band (62 kDa) resulting from still some L-TYR species contamination. Only faint bands were detected in the range of small molecular masses (∼15 kDa), corresponding to the well characterized protein *ab*Lectin (ABL, UniProt: Q00022, (Carrizo et al., 2005)) and some fragments of PPO4. ABL had been previously reported to be a contaminant in commercially available mushroom tyrosinase (Flurkey et al., 2008; Rescigno et al., 2007). No peptides corresponding to the in literature (Ismaya et al., 2011) described small subunit attached to A-TYR of PPO3 (H_2_L_2_) were found. Hence, it can be assumed that A-TYR of PPO4 might not have any affinity to this small subunit and a tetrameric structure (H_2_L_2_) might not be the case for PPO4.

The RP-HPLC–ESI-MS/MS based analysis of the tryptic peptides isolated from the 62 kDa band resulted in the unambiguous identification of the enzyme as polyphenol oxidase 4 (PPO4) from *A. bisporus* (C7FF05, UniProt.: PPO4) giving a score of 468 (−10lgP) and a sequence coverage of 95% (Table 1 and Fig. 3). Importantly, peptides outside the core region and belonging to the *C*-terminal part (Ser^383^–Phe^611^) of the protein could be assigned and verified by MS/MS fragmentation data (Fig. 3 and Table 1, peptides beneath the red line) giving evidence for the existence of PPO4 in a, by definition, latent form. No peptides of the protein region beyond Ala^574^ were found.

The ESI-QTOF-MS measurements gave evidence for the presence of five (sub)-species (A–E) of the intact PPO4 L-TYR, resulting from proteolytic fringing, exhibiting molecular masses of B = 64,247.3 Da; A = 64,034 Da; C = 64,546 Da; D = 64,647 Da; and E = 64,718 Da, respectively (Fig. 4). Calculating the theoretical mass of PPO4, including a thioether bridge (Cys^80^–His^82^) and accounting for the presence of an acetylated *N*-terminus, a disulfide bridge and the eight mutations (PPO4 → K9I869) an agreement with the experimental mass within an error of less than 1.9 Da is attained when assuming that the peptide backbone of the isolated protein ranges from Ser^2^–Thr^565^ from (M_r_ calc. = 64,249.2 Da). Moreover, by analyzing the mass gaps between all the mass spectrometric determined species (A–E) the *C*-terminal end of latent PPO4 could be assigned. Although a major species (B) ending with Thr^565^ does exist in an approximate ratio of 6:1 the *C*-terminal end seems to be rather fringed. This is additionally supported by the peptide mass analyses which found all kinds of possible *C*-terminal end peptides. However, the final 26 amino acid long *C*-terminal part appears to be proteolytically removed in any case (Fig. 3).

The eight in the above mass calculation included mutations at position Val^33^ → Leu, Ala^45^ → Ser, Val^101^ → Ile, Val^121^ → Ala, Ser^179^ → Asn, Arg^188^ → Lys, Ala^207^ → Gly and Leu^391^ → Gln can be found in the MS/MS analysis after tryptic digestion as being present together with the non-mutated peptides. The agreement between the molecular mass determined for species A–E in Fig. 4 and the calculated mass for the non-mutated form (Δ*m* = 15.95 Da) of PPO4 is matching with the preceding ∼16 Da shoulder, making it very likely that both forms, the non-mutated (PPO4) and the mutated (K9I869), are present simultaneously in our sample.

Reasonably, it can be noted, that certain sequence heterogeneity (differing strains) is given within our sample as lots of mushrooms from several boxes (differing mycelia individuals) were used for isolating the protein.

The derived mass spectra of A-TYR resulted in one single peak (other peaks are potassium adducts) with the mass of 43,673.1 Da. Due to fact that one of the sequence disparities (Leu^391^ → Gln, (Δ*m* = 14.97 Da) is placed on the *C*-terminal part of the protein the total mass difference between A-TYR (PPO4) and A-TYR (K9I869) is only 1.0 Da and is therefore not resolved in the spectrum. However, a final length of A-TYR is concluded to start with Ser^2^ and end with Ser^383^ (including acetylated *N*-termius and thioether bridge) giving an unexpected cleavage position since active PPO3 ends with the common YG-motif located four amino acids earlier (Ismaya et al., 2011).

Sequence alignment of all six *ab*PPO sequences shows a total identity between all isoforms of 11.83%, with the highest identity matches of 76.4% between PPO3 and PPO5 (Weijn et al., 2013). PPO4 is taxonomically most related to PPO2 with a sequence identity of 57.6%. The core region entwining and therefore stabilizing tyrosine-motif (Y-X-Y/F) is located at amino acid position 350–352, as well as the A-TYR ending YG-motif at position 378–379 (Fig. 3). No disulfide bond can occur between the *C*-terminal part and the core region (Ser^2^–Ser^383^), as the only cysteine in the core region is Cys^80^ which forms a thioether bond with His^82^. The two cysteines in the *C*-terminal part (Cys^462^, Cys^465^) are located on a peptide found in the HPLC–MS/MS based peptide analysis likely exhibiting a disulfide bound form (Fig. 3).

PPO4 contains 35 amino acids (corresponding to approx. 2 kDa) more than other *ab*PPO’s (except PPO6) (Weijn et al., 2013). Homology ends with Gly^563^ and the 48 amino acid long (4.2 kDa) final part of the *C*-terminus is unique for PPO4. This *C*-tail from Thr^565^ to Phe^611^ mainly consists of hydrophobic amino acids (*Grand average of hydropathicity*, GRAVY: 1.128). One can speculate that this might be an indication for a membrane related origin. Computational model calculations towards secondary structure prediction for membrane proteins yielded the results shown in Fig. 5, proposing that the peptide form an integral membrane helix between the amino acids Ala^569^ to Ala^591^. With such a hydrophobic structure, specifically PPO4, and unlike to the other PPOs, could be a membrane-bound protein. However, only a small part of this putative anchor (maximum four amino acids and not exceeding Ala^574^) was found in the isolated protein when analyzing the tryptic peptides by MS/MS. It is not likely that larger peptides out of this anchor were present in significant amounts but were overseen by the HPLC–ESI-MS/MS analysis, as the MS spectrum of the entire protein allows the accurate determination of the molecular mass of the major species which is in excellent agreement with a polypeptide chain ranging from Ser^2^ to Thr^565^. However, it cannot finally be excluded that the *C*-terminal tail was present *in vivo* and was removed, despite all protease inhibition efforts, during the early sample preparation and purification steps by proteolytic cleavage.

Regarding evidences for additional modifications, two potential sites for N-glycosylation are located at position Asn^349^ (core region) and Asn^441^ (*C*-terminal), respectively. In the HPLC–MS/MS based peptide analysis, the peptide containing the both consensus sequences (Asn^349^, Asn^441^) were found without glycosylation.

Further investigations are needed for clarifying in detail the activation process and mechanism. It is still not clear when and why the proteolytic cleavage of L-TYR happens and which proteases are responsible. Therefore, their characterization, identification, distinction and attribution of all isoforms are necessary. Also, the occurrence of protein complexes with small subunits, as was reported for PPO3, and which were definitely not found in our investigation, is not yet clarified neither in respect to their origin nor to function. In respect to all these research aims the elucidation of the L-TYR crystal structure would be of great benefit.

## Concluding remarks

Protein isolation and purification from natural sources, particularly from fungi or plants, in a manner that the proteins preserve their physiological constitution represents a methodological problem in biochemical research. In this study we present a technique capable to oppose the causes that are believed being the main source for protein alteration (e.g. formation of polyphenols and dying compounds). The study was focused on highly purifying mushroom tyrosinase a protein known as intricate to access. The method succeeded in isolating one out of six distinct isoforms (i.e. PPO4) and purifying it to identity. Notably, this optimized approach is applicable for many poorly accessible proteins from natural sources.

PPO4 could be identified and characterized in its latent precursor form by means of mass spectrometry displaying some PTMs, strain origin related sequence disparities and a somewhat fringed *C*-terminus. The exact cleavage position for the enzyme activation was determined to be located four amino acids behind the common tyrosinase YG-motif. Additionally, a membrane related origin could be assigned by computational methods.

## Experimental

All chemicals used were purchased from *Sigma–Aldrich* at highest available quality.

### Mushroom cultivation

Boxes containing fungal inoculated mulch covered with soil were used for cultivating white button mushroom. They were kept at constant temperature (15 °C) and humidity (95%). Mushrooms were occasionally harvested when the fruit-body reached growth-stage five (Hammond and Nichols, 1976). Fruit-body caps were discarded and the stipes were lyophilized for 3 days and grounded to powder for storage at room temperature.

### Pigment removal

Mushroom powder was suspended in a 125 mM sodium citrate buffer, pH 5.3, containing 35% (m/v) polyethylene glycol (PEG-4000), 25 mM sodium l-ascorbate, 20 mM l-lysine, 50 mM l-proline and 8% (v/v) triton X-114. Phenylmethylsulfonyl fluoride (PSMF) and benzamidine hydrochloride in dimethylsulfoxide (DMSO), to a final concentration of 1 mM for both, were added. The suspension was thoroughly mixed by an ultra turrax and subsequently centrifuged at 14,500 rpm (*Beckmann XP26*, rotor: JLA 16.250) for 10 min at 4 °C. The supernatant was discarded and the pellet resuspended in the above mentioned buffer. This mushroom powder extraction procedure was repeated twice. The obtained mushroom pulp, still containing high amounts of triton X-114 and PEG, was frozen at −80 °C for storage (optional).

### Protein extraction/purification

Frozen mushroom pulp was defrosted at 4 °C and suspended in a 125 mM sodium citrate buffer, pH 5.3, containing 25 mM sodium l-ascorbate, 20 mM l-lysine, 50 mM l-proline. Again, PSMF and benzamidine hydrochloride pre-solved in DMSO were added (final concentration 1 mM), as well as two protease inhibitor cocktails (P8215 0.1% (v/v) and S8830 1 tab/L). The suspension was stirred for 15 min and subsequently centrifuged (14,500 rpm at 4 °C over 10 min). The pellet and the highly viscose triton-phase, covering the pellet, were discarded. The supernatant was adjusted to 20% (113 g/L) ammonium sulfate saturation and centrifuged (14,500 rpm at 4 °C over 10 min). The occurring pellet was discarded and PEG-4000 was dissolved to a concentration of 15% (m/v) in the supernatant at 4 °C. After further centrifugation (14,500 rpm, 4 °C, 10 min) the generated PEG phase (upper phase) containing the non-target proteins was discarded. This step was followed by the subsequent addition of another 5% (m/v) of PEG-4000 and centrifugation. This last step was repeated twice. During the extraction/purification process every 45 min a protease inhibitor mixture containing PMSF, benzamidine hydrochloride and the two cocktails P8215/S8830 (*Sigma–Aldrich*) pre-dissolved in DMSO was added.

### Protein purification by fast protein liquid chromatography (FPLC)

All chromatographic purification steps were carried out using an *Äkta Purifier* (*GE Healthcare*) placed in a refrigerator to maintain 4 °C. In all chromatographic purification steps protein amounts were determined by setting the detection wavelengths to 280 nm (absorption of aromatic amino acids) and 345 nm (absorption of type-3 copper sites (Jolley et al., 1969)), respectively. The clear protein solution yielded by the steps described above was filtrated and loaded onto a size exclusion (SEC) column (Sephadex G-50, *GE Healthcare,* length = 30 cm, i.d. = 5 cm). A 30 mM HEPES buffer, pH 7.5, containing 10 mM l-lysine and 20 mM l-proline was used as elution buffer; the flow rate was 10 mL/min. The first eluted protein peak containing high molecular mass fraction was directly transferred on a straightly connected anion exchange column (DEAE–Sepharose FF, *GE Healthcare*, L = 20 cm, i.d. = 2.6 cm) equilibrated with a 30 mM HEPES buffer, pH 7.5. Proteins were eluted by stepwise increasing the sodium chloride concentration (1st step: 0.1 M (for 20 min), 2nd step: 0.2 M (for 25 min), 3rd step: 1 M (for 20 min)) at a constant flow rate of 4 mL/min. The protein fraction eluted in the 2nd step was collected and diluted to a conductivity below 5 mS/cm by a 20 mM Tris–HCl buffer pH 8.0 and directly loaded onto an anion exchange column (Q-Sepharose FF, *GE Healthcare*, L = 14 cm, i.d. = 2.6 cm) which was equilibrated analogously. Proteins were eluted by an increasing sodium chloride gradient depicted in Fig. 1A. Fractions were collected in a size of 12 mL and all collected fractions were tested photometrically for monophenolase activity (see enzymatic assay). Two isoforms were eluted at about 130 mM and 220 mM sodium chloride concentration, respectively. Fractions containing the first eluted protein isoform were pooled and ultra filtrated (size exclusion membrane of 30 kDa) centrifuged (4000 rpm) for removing sodium chloride. Then the protein was loaded on a cation exchange column (MonoS 5/50 GL, *GE Healthcare*) for further purification. Buffer conditions were 20 mM MES buffer at pH 5.5; proteins were eluted by an increasing sodium chloride gradient (0–0.13 M) with a very low slope (1.4 ML^−1^). While the active form of the enzyme (A-TYR) passed through the column three isoforms (L-TYR), #1, #2 and #3, were eluted at 11.5 mM, 13 mM and 14 mM sodium chloride concentration, respectively (Fig. 1B). This time, fractions of the first eluted protein, #1, were pooled and sodium chloride was removed via ultracentrifugation. The isolated fraction with #1 was further loaded (20 mM Tris–HCl, pH 9.0) onto an anion exchange column (MiniQ 4.6/50 PE, *GE Healthcare*) for removing further non-target proteins in a final polishing step (Fig. 1C). Again, elution was performed by an increasing sodium chloride gradient (20 mM Tris–HCl, pH 9.0, 0.5 M sodium chloride). The target-protein eluted at a 47 mM sodium chloride concentration. After this purification step characterization experiments for analyzing the sample composition were performed.

### Enzymatic assay

The enzymatic monophenolase activity was determined by monitoring the change in UV absorbance at 305 nm (dopachrome formation (Rodríguez-López et al., 1992)) (Fig. 1D). When measuring the activity of “active forms” of TYR, 10 μL of enzyme solution were added to 1 mL reaction mixture containing 35 mM potassium phosphate buffer, pH 6.5, and 0.033 mM tyrosine as substrate. One unit [U] monophenolase activity was defined as the increase of 1 mAU per minute at a path length of 1 cm at 25 °C. When measuring activity of the latent forms of the enzyme, the buffer system (for better solubility of SDS) was changed to 10 mM sodium phosphate buffer, pH 6.5, containing 1.3 mM SDS. Latency was calculated using the following equation.

$$\text{latency}\;[\%]=\left(1-\frac{\text{activity}(-\text{SDS})[\text{U}\;{\text{mL}}^{-1}]}{\text{activity}(+\text{SDS})[\text{U}\;{\text{mL}}^{-1}]}\right)*100$$

### Analytical polyacrylamide gel electrophoresis (PAGE)

Analyses by denaturating SDS–PAGE under reducing (2-mercaptoethanol) and non-reducing conditions (data for non-reducing not shown), respectively, were established as described elsewhere using a total polyacrylamide concentrations of 12% (Laemmli, 1970). Reduced samples were reacted with 3-iodopropionamide for alkylation of Cysteines. Sample load onto the gel was about 2 μg. Gels were stained with *Coomassie Brilliant Blue*. The target protein (L-TYR, 62 kDa) as well as the faint bands (∼15 kDa) in the A-TYR lane were cut out and used for protein identification. Imaging of the gels was done with the Gel Doc™ XR of *Bio Rad* (Fig. 2).

### Protein identification and sequence analysis

The gel piece covering the single bands obtained by denaturing SDS–PAGE (reduced and non-reduced) and containing approximately 2 μg of protein were used. Proteomic analyses were carried out after tryptic digestion by use of nanoLC–ESI-MS/MS with a LTQ-Orbitrap mass spectrometer. In addition samples were also measured by *Proteom Factory* AG (www.proteomefactory.com). Protein identification was based on the Mascot Search software and the NCBInr 110509 database as well as Peaks Studio 6.0 search software and UniProt/SwissProt database, respectively. Peptide mass tolerance was 5 ppm and fragment mass tolerance 0.5 Da. Variable modifications allowed were oxidation of methionine and, in the cases of reduced samples, also propionamidation of cysteines. For detailed information about the used method- and search software-parameters see the Supporting information.

### Determination of the molecular mass

The mass spectrum of the intact protein was measured using a nanoESI-QTOF mass spectrometer (maXis 4G UHR-TOF, *Bruker*) with a mass resolving power of about 40,000 in the used *m*/*z* – range and a mass accuracy of better than 5 ppm (confirmed by standard proteins). Prior to MS measurements, the purified L-TYR solution was ultra filtrated by centrifugation (10,000 rpm) and the buffer system was changed to 5 mM ammonium acetate pH 5.5 in order to reduce salt concentration to a minimum. Afterward acetonitrile (ACN, MS grade) and formic acid were added to a final concentration of 25% (v/v) ACN and 0.05% (v/v) formic acid. Sample introduction (1 μL sample volume contains about 0.3 μg of protein, taken from 20 μL total sample volume) was done by using a nano-spray robotic device (Nanomate, *Advion Biosciences*).

### Computational structure predictions

For the calculation of the GRAVY index (*Grand average of hydropathicity)* the free online software *ProtParam* provided by ExPASy.org was used. The secondary structure prediction for membrane proteins was done by using the *SOSUI* software engine (ver. 1.11) provided by the University of Nagoya (http://bp.nuap.nagoya-u.ac.jp/sosui). The sequence alignment was done by using the alignment program of uniprot.org.

## Figures and Tables

**Table 1:** List of peptides (reduced and non-reduced) found by nanoLC–ESI-MS/MS protein identification experiments of L-TYR (PPO4). Total −10lgP: 468. (Total score: 2604).^a^ Sequence coverage: 95%. Solely identified peptides with lowest divergence [ppm] are listed (For full data set see Supporting information). Peptides beneath the red line are *C*-terminal located.

a To some extend the sample was also evaluated by Proteome Factory AG (www.proteomefactory.com) giving an alternatively calculated score (Supporting information).
