# Supplementary material for: High level protein-purification allows the unambiguous polypeptide determination of latent isoform PPO4 of mushroom tyrosinase
Source: Phytochemistry. 2014 Mar;99(100):14–25. doi: 10.1016/j.phytochem.2013.12.016 (PMC3969299; doi:10.1016/j.phytochem.2013.12.016)
Supplement: Supplementary data 1 [file mmc1.docx]

**Supporting Information**

**High level protein-purification allows the unambiguous polypeptide determination of latent isoform PPO4 of mushroom tyrosinase.**

Stephan G. Mauracher *^a^*, Christian Molitor *^a^*, Claudia Michael *^b^*, Martin Kragl *^a^*, Andreas Rizzi *^b^* and Annette Rompel^*^ *^a^*

^a^ Mag. S. G. Mauracher, Dipl.- Chem. C. Molitor, Mag. M. Kragl, Prof. A. Rompel
Department of Biophysical Chemistry
University of Vienna
Althanstraße 14, 1090 Vienna (Austria)
Fax: +43 1 4277 9525
E-mail: annette.rompel@univie.ac.at

^b^ MSc. C. Michael, Prof. A. Rizzi
Department of Analytical Chemistry
University of Vienna
Währinger Straße 38, 1090 Vienna (Austria)

**Inventory:**

1. **Protein identification data**
2. **MS-data of intact protein (non-denaturating)**
3. **Basic calculated data (*i*P, M_r_) of PPO1-6**
4. **Protein identification data**

| **Reduced sample L-TYR; run 1 (measured by the Department of Analytical Chemistry)** | |
| --- | --- |
| Device and operating software used | Nano-HPLC (*Dionex Corporation*), Chromeleon Client Version 6.80 (*Dionex Corporation*); LTQ Orbitrap Velos (*Thermo Scientific*), LTQ Tune Plus Version 2.6.0 1065 SP3 (*Thermo Scientific*) |
| Software used (peak list generating) | Xcalibur 2.2 SP1.48 (*Thermo Scientific*) |
| Acquisition parameters | MS1 scan: m/z 400 – 1400; Filling time: 500 ms with 10^6^ ions; Resolution: 60.000; Fragmentation: CID with 35 eV; Peak picking: Top10 (intensity) with isolation window 3 m/z; Resolution; 7.500; Target ion previously selected for fragmentation were dynamically excluded for 180 s with relative mass window of 5 ppm. |
| **Search Parameters** | |
| Search engine | Peaks studio 6.0 |
| Enzyme specify | Trypsin |
| Number of miss cleavages permitted | 3 |
| Number of non-specific cleavage | 0 |
| Fixed modifications (including residue specificity) | 0 |
| Variable modifications (including residue specificity) | 0 |
| Mass tolerance for precursor ions | ±5 ppm |
| Mass tolerance for fragment ions | ±0.5 Dalton |
| Name of database searched (version/date) | UniProt (UniProtKB/Swiss-Prot, March 2013) |
| Species restriction and justification for searching only a subset of a database | no |
| Number of protein entries in the database actually searched | 35502518 |
| Cut-off score/expectation value for accepting individual MS/MS spectra provided | -10lgP ≥ 15 |
| Software used for PTM determination | Peaks Studio 6.0 |

Table 1a. Experimental settings for nano-HPLC ESI-MS/MS measurements and data evaluation for run 1. Sample: Tryptic digest of a gel band (L-TYR, 62 kDa) from a SDS-PAGE under reducing conditions.

| **Protein identification data (run 1)** | | | |
| --- | --- | --- | --- |
| Accession number | Unique peptides detected | Sequence coverage [%] | Score (-10lgP) |
| C7FF05\|PPO4_AGABI | 42 | 75 | 429.95 |
| P04264\|K2C1_HUMAN | 6 | 11 | 216.54 |
| P35527\|K1C9_HUMAN | 2 | 4 | 122.98 |
| Q6IG01\|K2C1B_RAT | 1 | 4 | 101.85 |
| P00761\|TRYP_PIG | 2 | 8 | 81.89 |

Table 2a. List of the five polypeptide sequences exhibiting the highest score for matching to the respective MS-data generated by run 1 (nano-HPLC ESI-MS/MS measurements).

| **Identified peptide list (run 1)** | | | | | | |
| --- | --- | --- | --- | --- | --- | --- |
|  | **Sequence** | **-10lgP** | **m (expected)** | **m/z (observed)** | **ppm** | **z** |
| 1 | M.SLLATVGPTGGVK.N | 52.03 | 1198.6921 | 600.3525 | -1.3 | 2 |
| 2 | K.NRLDIVDFVR.D | 83.21 | 1245.6830 | 623.8475 | -2.0 | 2 |
| 3 | R.LDIVDFVR.D | 76.22 | 975.5389 | 488.7769 | 0.3 | 2 |
| 4 | R.LDIVDFVRDEK.F | 75.50 | 1347.7034 | 674.8584 | -0.8 | 2 |
| 5 | K.FFTLYVR.A | 70.87 | 944.5120 | 473.2629 | -0.7 | 2 |
| 6 | R.ALQAIQDK.D | 47.02 | 885.4919 | 443.7524 | -1.8 | 2 |
| 7 | K.DQADYSSFFQLSGIHGLPFTPWAKPK.D | 90.42 | 2936.4497 | 735.1176 | -2.8 | 4 |
| 8 | R.VYVSIYEQVLQEAAK.G | 123.08 | 1738.9141 | 580.6452 | -0.1 | 3 |
| 9 | K.KEWVQAAEDLR.Q | 76.63 | 1343.6833 | 448.9016 | -0.1 | 3 |
| 10 | K.EWVQAAEDLR.Q | 23.79 | 1215.5884 | 608.7998 | -2.7 | 2 |
| 11 | R.QPYWDTGFALVPPDEIIK.L | 91.72 | 2088.0566 | 1045.0353 | -0.4 | 2 |
| 12 | K.ITNYDGTK.I | 47.79 | 910.4396 | 456.2277 | 1.5 | 2 |
| 13 | K.ITNYDGTKITVR.N | 35.41 | 1379.7408 | 460.9201 | -1.7 | 3 |
| 14 | R.NPILR.Y | 23.48 | 611.3755 | 612.3835 | 1.2 | 1 |
| 15 | R.YSFHPIDPSFSGYPNFDTWR.T | 44.97 | 2432.0862 | 1217.0459 | -3.7 | 2 |
| 16 | R.NPDADKKENIPALIAK.L | 60.50 | 1735.9468 | 434.9930 | -2.2 | 4 |
| 17 | K.KENIPALIAK.L | 52.34 | 1095.6652 | 548.8384 | -2.6 | 2 |
| 18 | K.ENIPALIAK.L | 51.16 | 967.5702 | 484.7921 | -0.4 | 2 |
| 19 | K.LDLEADSTR.E | 74.20 | 1018.4930 | 510.2535 | -0.5 | 2 |
| 20 | R.EKTYNMLK.F | 35.88 | 1025.5216 | 513.7672 | -1.7 | 2 |
| 21 | K.TYNMLK.F | 23.40 | 768.3840 | 769.3898 | -1.9 | 1 |
| 22 | K.FNANWEAFSNHGEFDDTHANSLEAVHDDIHGFVGR.G | 78.77 | 3954.7371 | 989.6899 | -1.6 | 4 |
| 23 | R.GHMTHALFAAFDPIFWLHHSNVDR.H | 108.56 | 2818.3550 | 564.6787 | 0.8 | 5 |
| 24 | R.HLSLWQALYPGVWVTQGPER.E | 119.27 | 2336.2065 | 779.7427 | -0.1 | 3 |
| 25 | R.EGSMGFAPGTELNK.D | 87.83 | 1436.6605 | 719.3362 | -1.9 | 2 |
| 26 | R.EGSMGFAPGTELNKDSALEPFYETEDKPWTSVPLTDTALLNYSYPDFDK.V | 48.97 | 5485.5386 | 1098.1168 | 1.7 | 5 |
| 27 | K.DSALEPFYETEDKPWTSVPLTDTALLNYSYPDFDKVK.G | 67.68 | 4294.0522 | 1074.5210 | 0.6 | 4 |
| 28 | K.DSALEPFYETEDKPWTSVPLTDTALLNYSYPDFDK.V | 65.53 | 4066.8887 | 1017.7263 | -3.1 | 4 |
| 29 | K.GGTPDLVR.D | 42.75 | 813.4344 | 814.4399 | -2.2 | 1 |
| 30 | R.DYINDHIDR.R | 72.13 | 1159.5258 | 580.7695 | -1.1 | 2 |
| 31 | R.DYINDHIDRR.Y | 55.17 | 1315.6268 | 658.8185 | -3.2 | 2 |
| 32 | K.SEGGKNPALDLLSDFK.G | 120.53 | 1689.8573 | 845.9344 | -1.8 | 2 |
| 33 | K.NPALDLLSDFK.G | 109.55 | 1231.6448 | 616.8287 | -1.5 | 2 |
| 34 | K.GVTHDHNEDLK.M | 45.30 | 1263.5844 | 632.8015 | 3.3 | 2 |
| 35 | K.MFDWTIQASWK.K | 95.70 | 1411.6594 | 706.8371 | 0.2 | 2 |
| 36 | K.KFELDDSFAIIFYFAADGSTNVTK.E | 93.45 | 2698.3167 | 900.4465 | 0.4 | 3 |
| 37 | K.ENYIGSINIFR.G | 85.62 | 1324.6775 | 663.3454 | -0.9 | 2 |
| 38 | R.TQDNLVQEGFVHLDR.F | 119.35 | 1769.8696 | 885.9407 | -1.6 | 2 |
| 39 | R.DLDTFDPQAVHR.Y | 99.75 | 1412.6685 | 707.3412 | -0.4 | 2 |
| 40 | K.VVADDHSVTLK.S | 74.56 | 1182.6244 | 592.3190 | -0.8 | 2 |
| 41 | K.VVADDHSVTLKSLR.I | 47.91 | 1538.8417 | 513.9543 | -0.2 | 3 |
| 42 | R.VQGRPLHLPPGVSFPR.L | 33.17 | 1755.9896 | 440.0044 | -0.6 | 4 |

Table 3a. List of peptides matches found by nano-HPLC ESI-MS/MS measurements for run 1.

| **Reduced sample L-TYR; run 2 (measured by the Department of Analytical Chemistry)** | |
| --- | --- |
| Device and operating software used | Nano-HPLC (*Dionex Corporation*), Chromeleon Client Version 6.80 (*Dionex Corporation*); LTQ Orbitrap Velos (*Thermo Scientific*), LTQ Tune Plus Version 2.6.0 1065 SP3 (*Thermo Scientific*) |
| Software used (peak list generating) | Xcalibur 2.2 SP1.48 (*Thermo Scientific*) |
| Acquisition parameters | MS1 scan: m/z 400 – 1400; Filling time: 500 ms with 10^6^ ions; Resolution: 60.000; Fragmentation: CID with 35 eV; Peak picking: Top10 (intensity) with isolation window 3 m/z; Resolution; 7.500; Target ion previously selected for fragmentation were dynamically excluded for 180 s with relative mass window of 5 ppm. |
| **Search Parameters** | |
| Search engine | Peaks studio 6.0 |
| Enzyme specify | Trypsin |
| Number of miss cleavages permitted | 3 |
| Number of non-specific cleavage | 1 |
| Fixed modifications (including residue specificity) | 0 |
| Variable modifications (including residue specificity) | 0 |
| Mass tolerance for precursor ions | ±5 ppm |
| Mass tolerance for fragment ions | ±0.5 Dalton |
| Name of database searched (version/date) | UniProt (UniProtKB/Swiss-Prot, March 2013) |
| Species restriction and justification for searching only a subset of a database | no |
| Number of protein entries in the database actually searched | 35502518 |
| Cut-off score/expectation value for accepting individual MS/MS spectra provided | -10lgP ≥ 15 |
| Software used for PTM determination | Peaks Studio 6.0 |

Table 4a. Experimental settings for nano-HPLC ESI-MS/MS measurements and data evaluation for run 2. Sample: Tryptic digest of a gel band (L-TYR, 62 kDa) from a SDS-PAGE under reducing conditions.

| **Protein identification data (run 2)** | | | |
| --- | --- | --- | --- |
| Accession number | Unique peptides detected | Sequence coverage [%] | Score (-10lgP) |
| C7FF05\|PPO4_AGABI | 212 | 84 | 468.36 |
| P04264\|K2C1_HUMAN | 8 | 13 | 172.83 |
| P00761\|TRYP_PIG | 7 | 28 | 132.41 |
| C7FF04\|PPO3_AGABI | 4 | 5 | 109.44 |
| P35527\|K1C9_HUMAN | 3 | 5 | 105.60 |

Table 5a. List of the five polypeptide sequences exhibiting the highest score for matching to the respective MS-data generated by run 2(nano-HPLC ESI-MS/MS measurements).

| **Identified peptide list (run 2)** | | | | | | |
| --- | --- | --- | --- | --- | --- | --- |
|  | **Sequence** | **-10lgP** | **m (expected)** | **m/z (observed)** | **ppm** | **z** |
| 1 | M.SLLATVGPTGGVK.N | 42.23 | 1198.6921 | 600.3524 | -1.6 | 2 |
| 2 | S.LLATVGPTGGVK.N | 30.44 | 1111.6600 | 556.8363 | -1.8 | 2 |
| 3 | L.LATVGPTGGVK.N | 21.98 | 998.5760 | 500.2944 | -1.8 | 2 |
| 4 | L.ATVGPTGGVK.N | 32.03 | 885.4919 | 443.7546 | 3.0 | 2 |
| 5 | K.NRLDIVDFVR.D | 64.94 | 1245.6830 | 623.8475 | -2.0 | 2 |
| 6 | N.RLDIVDFVR.D | 44.96 | 1131.6400 | 566.8270 | -0.5 | 2 |
| 7 | R.LDIVDFVRDEK.F | 62.44 | 1347.7034 | 674.8584 | -0.8 | 2 |
| 8 | R.LDIVDFVR.D | 54.45 | 975.5389 | 488.7765 | -0.4 | 2 |
| 9 | L.DIVDFVR.D | 26.83 | 862.4548 | 863.4602 | -2.2 | 1 |
| 10 | D.IVDFVR.D | 17.53 | 747.4279 | 748.4336 | -2.1 | 1 |
| 11 | R.ALQAIQDK.D | 38.12 | 885.4919 | 886.4982 | -1.2 | 1 |
| 12 | R.ALQAIQDKD.Q | 34.22 | 1000.5189 | 501.2663 | -0.9 | 2 |
| 13 | R.ALQAIQDKDQADYSSFFQL.S | 56.26 | 2187.0483 | 730.0224 | -1.3 | 3 |
| 14 | R.ALQAIQDKDQADYSSFFQLSGIHGLPFTPWAK.P | 82.46 | 3578.7832 | 895.7042 | 1.3 | 4 |
| 15 | R.ALQAIQDKDQADYSSFFQLSGIHGLPF.T | 44.81 | 2995.4714 | 999.4998 | 2.1 | 3 |
| 16 | R.ALQAIQDKDQADYSSFFQLSGIHGLPFTPW.A | 53.53 | 3379.6514 | 845.9191 | -1.2 | 4 |
| 17 | A.LQAIQDK.D | 17.06 | 814.4548 | 408.2337 | -2.3 | 2 |
| 18 | K.DQADYSSFFQLSGIH.G | 71.84 | 1713.7634 | 857.8887 | -0.3 | 2 |
| 19 | K.DQADYSSFFQLSGIHGLPFTPWAKPK.D | 71.49 | 2936.4497 | 735.1176 | -2.8 | 4 |
| 20 | K.DQADYSSFFQLS.G | 44.10 | 1406.5990 | 704.3080 | 1.8 | 2 |
| 21 | K.DQADYSSFFQLSGIHGLPFTPWAK.P | 80.30 | 2711.3020 | 904.7749 | 0.3 | 3 |
| 22 | K.DQADYSSFFQL.S | 48.66 | 1319.5669 | 660.7911 | 0.6 | 2 |
| 23 | K.DQADYSSFFQLSGIHGLPF.T | 65.51 | 2127.9900 | 710.3370 | -0.3 | 3 |
| 24 | K.DQADYSSFFQLSGIHGLPFTPW.A | 66.45 | 2512.1699 | 838.3985 | 1.5 | 3 |
| 25 | K.DTPTVPY.E | 26.24 | 791.3701 | 792.3765 | -1.2 | 1 |
| 26 | K.DTPTVPYESGY.C | 48.63 | 1227.5295 | 1228.5359 | -0.8 | 1 |
| 27 | D.TPTVPYESGYCTHSQVLFPTWHR.V | 19.36 | 2705.2695 | 677.3215 | -4.6 | 4 |
| 28 | H.SQVLFPTWHR.V | 48.90 | 1269.6619 | 635.8378 | -0.7 | 2 |
| 29 | L.FPTWHR.V | 25.16 | 842.4188 | 422.2164 | -0.5 | 2 |
| 30 | R.VYVSIYEQ.V | 39.94 | 999.4913 | 500.7525 | -0.7 | 2 |
| 31 | R.VYVSIYEQVLQEAAK.G | 88.18 | 1738.9141 | 580.6452 | -0.1 | 3 |
| 32 | R.VYVSIYEQVLQEA.A | 60.97 | 1539.7820 | 770.8968 | -1.9 | 2 |
| 33 | Y.VSIYEQVLQEAAK.G | 68.30 | 1476.7823 | 739.3976 | -1.2 | 2 |
| 34 | K.KEWVQAAEDLR.Q | 55.19 | 1343.6833 | 448.9016 | -0.1 | 3 |
| 35 | K.EWVQAAEDLR.Q | 21.91 | 1215.5884 | 608.7998 | -2.7 | 2 |
| 36 | R.QPYWDTGF.A | 31.49 | 1012.4290 | 507.2216 | -0.4 | 2 |
| 37 | R.QPYWDTGFALVPPDEIIK.L | 63.17 | 2088.0566 | 1045.0349 | -0.7 | 2 |
| 38 | R.QPYWDTGFALVPPD.E | 27.16 | 1604.7511 | 803.3833 | 0.6 | 2 |
| 39 | Q.PYWDTGFALVPPDEIIK.L | 69.44 | 1959.9982 | 981.0062 | -0.1 | 2 |
| 40 | P.YWDTGFALVPPDEIIK.L | 65.27 | 1862.9454 | 932.4794 | -0.6 | 2 |
| 41 | Y.WDTGFALVPPDEIIK.L | 58.13 | 1699.8821 | 850.9488 | 0.6 | 2 |
| 42 | D.TGFALVPPDEIIK.L | 43.14 | 1398.7758 | 700.3940 | -1.7 | 2 |
| 43 | G.FALVPPDEIIK.L | 36.28 | 1240.7067 | 621.3603 | -0.5 | 2 |
| 44 | F.ALVPPDEIIK.L | 23.02 | 1093.6383 | 547.8254 | -1.9 | 2 |
| 45 | V.PPDEIIK.L | 19.68 | 810.4487 | 811.4553 | -0.8 | 1 |
| 46 | K.ITNYDGTK.I | 43.35 | 910.4396 | 456.2277 | 1.5 | 2 |
| 47 | K.ITNYDGTKITVR.N | 33.45 | 1379.7408 | 460.9201 | -1.7 | 3 |
| 48 | R.YSFHPID.P | 40.76 | 877.3970 | 439.7052 | -1.3 | 2 |
| 49 | R.YSFHPIDPSF.S | 41.56 | 1208.5502 | 605.2822 | -0.3 | 2 |
| 50 | R.YSFHPIDPSFSGYPNFDTWR.T | 43.53 | 2432.0862 | 1217.0459 | -3.7 | 2 |
| 51 | S.GYPNFDTWR.T | 41.81 | 1154.5145 | 578.2642 | -0.5 | 2 |
| 52 | R.NPDADKKENIPALIAK.L | 46.31 | 1735.9468 | 434.9930 | -2.2 | 4 |
| 53 | K.KENIPALIAK.L | 43.95 | 1095.6652 | 548.8384 | -2.6 | 2 |
| 54 | K.ENIPALIAK.L | 38.46 | 967.5702 | 968.5763 | -1.2 | 1 |
| 55 | A.KLDLEADSTR.E | 42.03 | 1146.5880 | 574.2997 | -2.7 | 2 |
| 56 | K.LDLEADSTR.E | 58.68 | 1018.4930 | 510.2535 | -0.5 | 2 |
| 57 | L.DLEADSTR.E | 33.67 | 905.4090 | 906.4149 | -1.5 | 1 |
| 58 | D.LEADSTR.E | 28.07 | 790.3821 | 791.3883 | -1.3 | 1 |
| 59 | R.EKTYNMLK.F | 32.13 | 1025.5216 | 513.7672 | -1.7 | 2 |
| 60 | K.TYNMLK.F | 21.69 | 768.3840 | 769.3907 | -0.7 | 1 |
| 61 | K.FNANWEAFSNHG.E | 50.07 | 1392.5847 | 697.2982 | -2.0 | 2 |
| 62 | K.FNANWEAFSNHGEFDDTHAN.S | 63.10 | 2321.9363 | 1161.9741 | -1.2 | 2 |
| 63 | K.FNANWEAFSNH.G | 46.79 | 1335.5632 | 668.7876 | -1.9 | 2 |
| 64 | K.FNANWEAFSNHGEFDDTHANSLEAVHDD.I | 47.69 | 3188.3132 | 798.0862 | 0.8 | 4 |
| 65 | K.FNANWEAFSNHGEFDDTHANSLEAVHD.D | 57.46 | 3073.2861 | 769.3282 | -0.8 | 4 |
| 66 | K.FNANWEAFSNHGEFDDTHANSLEAVHDDIHGFVGR.G | 70.88 | 3954.7371 | 989.6899 | -1.6 | 4 |
| 67 | K.FNANWEAFSNHGEFDDTHANSL.E | 66.46 | 2522.0522 | 841.6911 | -0.3 | 3 |
| 68 | K.FNANWEAFSNHGEFDDTHANSLEAVHDDIHGF.V | 57.53 | 3642.5461 | 911.6433 | -0.5 | 4 |
| 69 | K.FNANWEAFSNHGEFD.D | 57.41 | 1783.7227 | 892.8680 | -0.7 | 2 |
| 70 | K.FNANWEAF.S | 32.52 | 997.4293 | 998.4347 | -1.9 | 1 |
| 71 | K.FNANWEAFSN.H | 31.48 | 1198.5043 | 600.2612 | 3.1 | 2 |
| 72 | N.ANWEAFSNHGEFDDTHANSLEAVHDDIHGFVGR.G | 48.94 | 3693.6257 | 739.7322 | -0.2 | 5 |
| 73 | N.WEAFSNHGEFDDTHANSLEAVHDDIHGFVGR.G | 63.49 | 3508.5457 | 702.7159 | -0.7 | 5 |
| 74 | F.SNHGEFDDTHANSLEAVHDDIHGFVGR.G | 89.01 | 2975.3181 | 744.8365 | -0.3 | 4 |
| 75 | N.HGEFDDTHANSLEAVHDDIHGFVGR.G | 100.83 | 2774.2432 | 694.5679 | -0.2 | 4 |
| 76 | D.DTHANSLEAVHDDIHGFVGR.G | 94.80 | 2189.0249 | 730.6805 | -2.4 | 3 |
| 77 | D.THANSLEAVHDDIHGFVGR.G | 94.57 | 2073.9980 | 692.3391 | -1.2 | 3 |
| 78 | N.SLEAVHDDIHGFVGR.G | 77.41 | 1650.8114 | 551.2777 | 0.0 | 3 |
| 79 | S.LEAVHDDIHGFVGR.G | 36.24 | 1563.7793 | 522.2657 | -2.6 | 3 |
| 80 | L.EAVHDDIHGFVGR.G | 78.87 | 1450.6953 | 726.3539 | -1.4 | 2 |
| 81 | D.DIHGFVGR.G | 38.93 | 899.4613 | 450.7374 | -1.2 | 2 |
| 82 | R.GHMTHALF.A | 47.18 | 912.4276 | 913.4343 | -0.7 | 1 |
| 83 | R.GHMTHALFA.A | 37.29 | 983.4647 | 492.7394 | -0.5 | 2 |
| 84 | R.GHMTHALFAAFD.P | 49.69 | 1316.5972 | 659.3046 | -1.9 | 2 |
| 85 | R.GHMTHALFAAFDPIFWLHHSNVDR.H | 87.81 | 2818.3550 | 564.6772 | -1.8 | 5 |
| 86 | H.MTHALFAAFDPIFWLHHSNVDR.H | 68.89 | 2624.2747 | 525.8625 | 0.7 | 5 |
| 87 | H.ALFAAFDPIFWLHHSNVDR.H | 80.19 | 2255.1274 | 1128.5703 | -0.6 | 2 |
| 88 | A.LFAAFDPIFWLHHSNVDR.H | 41.26 | 2184.0903 | 547.0287 | -2.0 | 4 |
| 89 | L.FAAFDPIFWLHHSNVDR.H | 66.94 | 2071.0063 | 518.7588 | -0.1 | 4 |
| 90 | F.AAFDPIFWLHHSNVDR.H | 65.52 | 1923.9380 | 642.3206 | 1.0 | 3 |
| 91 | A.AFDPIFWLHHSNVDR.H | 47.23 | 1852.9009 | 464.2321 | -0.7 | 4 |
| 92 | A.FDPIFWLHHSNVDR.H | 45.49 | 1781.8638 | 446.4728 | -0.9 | 4 |
| 93 | D.PIFWLHHSNVDR.H | 67.48 | 1519.7684 | 760.8910 | -0.6 | 2 |
| 94 | R.HLSLWQ.A | 18.23 | 782.4075 | 783.4142 | -0.8 | 1 |
| 95 | R.HLSLWQA.L | 33.65 | 853.4446 | 854.4515 | -0.4 | 1 |
| 96 | R.HLSLWQALYPGVWVTQGPER.E | 91.94 | 2336.2065 | 779.7427 | -0.1 | 3 |
| 97 | R.HLSLWQALYPGVWVTQ.G | 55.57 | 1896.9886 | 949.5019 | 0.3 | 2 |
| 98 | R.HLSLWQALYPGVWVT.Q | 53.79 | 1768.9301 | 885.4719 | -0.5 | 2 |
| 99 | L.WQALYPGVWVTQGPER.E | 63.30 | 1885.9475 | 943.9799 | -1.2 | 2 |
| 100 | W.QALYPGVWVTQGPER.E | 66.11 | 1699.8682 | 850.9415 | 0.2 | 2 |
| 101 | Q.ALYPGVWVTQGPER.E | 69.04 | 1571.8096 | 786.9106 | -1.9 | 2 |
| 102 | A.LYPGVWVTQGPER.E | 69.39 | 1500.7725 | 751.3933 | -0.2 | 2 |
| 103 | L.YPGVWVTQGPER.E | 65.12 | 1387.6885 | 694.8508 | -1.1 | 2 |
| 104 | Y.PGVWVTQGPER.E | 62.99 | 1224.6251 | 613.3197 | -0.2 | 2 |
| 105 | R.EGSMGFAPGTELNK.D | 76.19 | 1436.6605 | 719.3362 | -1.9 | 2 |
| 106 | R.EGSMGFAPGTELNKD.S | 60.63 | 1551.6875 | 776.8492 | -2.4 | 2 |
| 107 | R.EGSMGFAPGTELNKDSALEPFYETEDKPWTSVPLTDTALLNYSYPDFDK.V | 43.70 | 5485.5386 | 1098.1168 | 1.7 | 5 |
| 108 | R.EGSMGFAPGTELNKDSALEPFYETEDKPWTSVPLTDTALLNY.S | 30.75 | 4633.1733 | 1159.3053 | 4.0 | 4 |
| 109 | E.GSMGFAPGTELNK.D | 49.51 | 1307.6179 | 654.8148 | -2.2 | 2 |
| 110 | G.SMGFAPGTELNK.D | 58.93 | 1250.5964 | 626.3041 | -2.1 | 2 |
| 111 | S.MGFAPGTELNK.D | 56.90 | 1163.5645 | 582.7885 | -1.7 | 2 |
| 112 | M.GFAPGTELNK.D | 44.07 | 1032.5239 | 517.2684 | -1.7 | 2 |
| 113 | G.FAPGTELNK.D | 43.77 | 975.5025 | 488.7581 | -0.9 | 2 |
| 114 | N.KDSALEPFYETEDKPWTSVPLTDTALLNYSYPDFDK.V | 41.63 | 4194.9834 | 1049.7538 | 0.6 | 4 |
| 115 | K.DSALEPFYETEDK.P | 66.16 | 1542.6725 | 772.3429 | -0.8 | 2 |
| 116 | K.DSALEPF.Y | 15.75 | 777.3544 | 778.3608 | -1.2 | 1 |
| 117 | K.DSALEPFYETEDKPWT.S | 69.58 | 1926.8523 | 964.4321 | -1.4 | 2 |
| 118 | K.DSALEPFYETED.K | 46.41 | 1414.5775 | 708.2949 | -1.6 | 2 |
| 119 | K.DSALEPFYETEDKPW.T | 66.29 | 1825.8046 | 913.9080 | -1.7 | 2 |
| 120 | K.DSALEPFYETEDKPWTSVPLTDTA.L | 62.12 | 2711.2490 | 904.7565 | -0.5 | 3 |
| 121 | K.DSALEPFYETEDKPWTSVPLTD.T | 58.40 | 2539.1641 | 847.3951 | -0.2 | 3 |
| 122 | K.DSALEPFYETEDKPWTSVPLTDTALLNYSYPDFDKVK.G | 59.05 | 4294.0522 | 1074.5210 | 0.6 | 4 |
| 123 | K.DSALEPFYETEDKPWTSVPL.T | 50.68 | 2323.0896 | 1162.5533 | 1.1 | 2 |
| 124 | K.DSALEPFYETEDKPWTSVPLTDTAL.L | 64.72 | 2824.3330 | 942.4531 | 1.6 | 3 |
| 125 | K.DSALEPFYETEDKPWTSVPLTDTALLNY.S | 70.44 | 3214.5234 | 1072.5145 | -0.6 | 3 |
| 126 | K.DSALEPFYETEDKPWTSVPLTDTALL.N | 65.88 | 2937.4170 | 980.1472 | 0.9 | 3 |
| 127 | K.DSALEPFYETEDKPWTSVPLTDTALLNYSYPD.F | 57.46 | 3676.6985 | 1226.5736 | 0.1 | 3 |
| 128 | K.DSALEPFYETEDKPWTSVPLTDTALLNYSYPDFD.K | 46.65 | 3938.7937 | 1313.9362 | -1.9 | 3 |
| 129 | K.DSALEPFYETEDKPWTSVPLTDTALLNYSYPDFDK.V | 62.53 | 4066.8887 | 1017.7305 | 1.0 | 4 |
| 130 | D.SALEPFYETEDKPWTSVPLTDTALLNYSYPDFDK.V | 62.22 | 3951.8618 | 988.9728 | 0.1 | 4 |
| 131 | F.YETEDKPWTSVPLTDTALLNYSYPDFDK.V | 67.56 | 3307.5447 | 1103.5190 | -2.9 | 3 |
| 132 | E.DKPWTSVPLTDTALLNYSYPDFDK.V | 71.25 | 2785.3486 | 929.4562 | -0.7 | 3 |
| 133 | D.KPWTSVPLTDTALLNYSYPDFDK.V | 84.68 | 2670.3218 | 891.1132 | -1.5 | 3 |
| 134 | K.PWTSVPLTDTALLNYSYPDFDK.V | 76.01 | 2542.2268 | 848.4162 | 0.0 | 3 |
| 135 | W.TSVPLTDTALLNYSYPDFDK.V | 44.50 | 2259.0947 | 754.0377 | -1.5 | 3 |
| 136 | T.SVPLTDTALLNYSYPDFDK.V | 68.39 | 2158.0469 | 1080.0310 | 0.2 | 2 |
| 137 | L.TDTALLNYSYPDFDK.V | 63.02 | 1761.8097 | 881.9110 | -1.2 | 2 |
| 138 | D.TALLNYSYPDFDK.V | 69.96 | 1545.7351 | 773.8748 | 0.0 | 2 |
| 139 | A.LLNYSYPDFDK.V | 61.36 | 1373.6503 | 687.8318 | -0.9 | 2 |
| 140 | L.LNYSYPDFDK.V | 58.56 | 1260.5662 | 631.2896 | -1.3 | 2 |
| 141 | L.NYSYPDFDK.V | 40.75 | 1147.4822 | 574.7478 | -1.0 | 2 |
| 142 | N.YSYPDFDKVK.G | 40.26 | 1260.6025 | 421.2079 | -0.5 | 3 |
| 143 | N.YSYPDFDK.V | 47.31 | 1033.4392 | 517.7266 | -0.6 | 2 |
| 144 | Y.SYPDFDK.V | 28.92 | 870.3759 | 871.3824 | -0.8 | 1 |
| 145 | V.KGGTPDLVR.D | 23.61 | 941.5294 | 471.7730 | 2.2 | 2 |
| 146 | K.GGTPDLVR.D | 38.51 | 813.4344 | 814.4399 | -2.2 | 1 |
| 147 | K.GGTPDLVRD.Y | 27.40 | 928.4614 | 465.2382 | 0.5 | 2 |
| 148 | R.DYINDHIDRR.Y | 47.97 | 1315.6268 | 658.8185 | -3.2 | 2 |
| 149 | R.DYINDHIDR.R | 61.57 | 1159.5258 | 580.7695 | -1.1 | 2 |
| 150 | R.DYINDHID.R | 32.15 | 1003.4247 | 502.7195 | -0.2 | 2 |
| 151 | D.YINDHIDR.R | 46.71 | 1044.4988 | 523.2564 | -0.5 | 2 |
| 152 | Y.INDHIDR.R | 16.93 | 881.4355 | 441.7246 | -0.9 | 2 |
| 153 | K.SEGGKNPALDLLSDFK.G | 88.81 | 1689.8573 | 845.9344 | -1.8 | 2 |
| 154 | K.NPALDLLSDFK.G | 79.37 | 1231.6448 | 616.8283 | -2.2 | 2 |
| 155 | K.NPALDLLSD.F | 33.45 | 956.4814 | 479.2481 | 0.2 | 2 |
| 156 | N.PALDLLSDFK.G | 56.94 | 1117.6019 | 559.8078 | -0.8 | 2 |
| 157 | P.ALDLLSDFK.G | 39.42 | 1020.5491 | 1021.5551 | -1.3 | 1 |
| 158 | K.GVTHDHNEDLK.M | 37.14 | 1263.5844 | 632.8018 | 3.7 | 2 |
| 159 | K.MFDWTIQASWK.K | 81.54 | 1411.6594 | 706.8365 | -0.6 | 2 |
| 160 | K.MFDWTIQAS.W | 48.66 | 1097.4852 | 549.7496 | -0.6 | 2 |
| 161 | K.MFDWTIQ.A | 18.75 | 939.4160 | 470.7155 | 0.4 | 2 |
| 162 | K.KFELDDSF.A | 33.65 | 999.4549 | 500.7343 | -0.7 | 2 |
| 163 | K.KFELDDSFAIIFYFAADGSTNVTK.E | 82.14 | 2698.3167 | 900.4465 | 0.4 | 3 |
| 164 | K.KFELDDSFAIIF.Y | 32.68 | 1443.7285 | 722.8711 | -0.6 | 2 |
| 165 | K.KFELDDSFAIIFY.F | 49.33 | 1606.7919 | 804.4036 | 0.5 | 2 |
| 166 | K.FELDDSFAIIFY.F | 59.68 | 1478.6969 | 740.3552 | -0.7 | 2 |
| 167 | F.YFAADGSTNVTK.E | 71.71 | 1272.5986 | 637.3057 | -1.4 | 2 |
| 168 | Y.FAADGSTNVTK.E | 50.35 | 1109.5353 | 555.7753 | 0.8 | 2 |
| 169 | F.AADGSTNVTK.E | 32.86 | 962.4669 | 482.2422 | 3.2 | 2 |
| 170 | K.ENYIGSIN.I | 29.05 | 908.4240 | 455.2185 | -1.7 | 2 |
| 171 | K.ENYIGSINIFR.G | 63.54 | 1324.6775 | 663.3457 | -0.5 | 2 |
| 172 | N.YIGSINIFR.G | 48.76 | 1081.5920 | 541.8026 | -1.2 | 2 |
| 173 | R.TQDNLVQEGFVH.L | 64.06 | 1385.6575 | 693.8351 | -1.2 | 2 |
| 174 | R.TQDNLVQEGFVHLDR.F | 89.57 | 1769.8696 | 885.9407 | -1.6 | 2 |
| 175 | R.TQDNLVQEGF.V | 41.38 | 1149.5302 | 1150.5364 | -1.0 | 1 |
| 176 | R.TQDNLVQEGFVHLD.R | 69.93 | 1613.7686 | 807.8899 | -2.0 | 2 |
| 177 | R.TQDNLVQEGFVHLDRF.I | 52.33 | 1916.9380 | 639.9856 | -1.5 | 3 |
| 178 | Q.DNLVQEGFVHLDR.F | 37.00 | 1540.7634 | 514.5947 | -0.7 | 3 |
| 179 | D.NLVQEGFVHLDR.F | 69.29 | 1425.7365 | 713.8754 | -0.2 | 2 |
| 180 | N.LVQEGFVHLDR.F | 60.40 | 1311.6935 | 656.8535 | -0.7 | 2 |
| 181 | V.QEGFVHLDR.F | 23.53 | 1099.5410 | 550.7776 | -0.2 | 2 |
| 182 | R.DLDTFDPQAVHR.Y | 81.11 | 1412.6685 | 707.3412 | -0.4 | 2 |
| 183 | D.LDTFDPQAVHR.Y | 68.43 | 1297.6415 | 649.8275 | -0.8 | 2 |
| 184 | L.DTFDPQAVHR.Y | 50.89 | 1184.5574 | 593.2855 | -0.8 | 2 |
| 185 | D.TFDPQAVHR.Y | 48.80 | 1069.5305 | 535.7710 | -2.8 | 2 |
| 186 | T.FDPQAVHR.Y | 33.53 | 968.4828 | 485.2475 | -2.3 | 2 |
| 187 | K.VVADDHSVTLK.S | 69.87 | 1182.6244 | 592.3188 | -1.0 | 2 |
| 188 | K.VVADDHSVTLKSLR.I | 43.78 | 1538.8417 | 513.9543 | -0.2 | 3 |
| 189 | K.VVADDHSVTL.K | 31.91 | 1054.5294 | 528.2722 | 0.3 | 2 |
| 190 | V.VADDHSVTLK.S | 34.30 | 1083.5560 | 542.7853 | 0.0 | 2 |
| 191 | V.ADDHSVTLK.S | 40.97 | 984.4876 | 493.2507 | -0.7 | 2 |
| 192 | A.DDHSVTLK.S | 15.42 | 913.4505 | 457.7314 | -2.3 | 2 |
| 193 | D.DHSVTLK.S | 16.26 | 798.4236 | 400.2172 | -4.7 | 2 |
| 194 | R.VQGRPLHLPPGVSFPR.L | 32.67 | 1755.9896 | 440.0044 | -0.6 | 4 |
| 195 | R.PLHLPPGVSFPR.L | 61.30 | 1315.7400 | 439.5867 | -1.2 | 3 |
| 196 | L.HLPPGVSFPR.L | 36.55 | 1105.6033 | 553.8077 | -2.2 | 2 |
| 197 | L.PPGVSFPR.L | 37.11 | 855.4603 | 428.7372 | -0.4 | 2 |
| 198 | K.NIPIVNFD.D | 44.17 | 930.4811 | 466.2473 | -1.0 | 2 |
| 199 | K.NIPIVNFDDVLD.L | 59.95 | 1372.6874 | 687.3510 | 0.0 | 2 |
| 200 | K.NIPIVNFDDVLDLVTGVVN.I | 62.20 | 2055.0889 | 686.0361 | -1.1 | 3 |
| 201 | K.NIPIVNFDDVLDLVTGVV.N | 49.68 | 1941.0459 | 971.5297 | -0.6 | 2 |
| 202 | K.NIPIVNFDDVLDLVTGVVNIG.L | 68.36 | 2225.1943 | 1113.6040 | -0.4 | 2 |
| 203 | K.NIPIVNFDDVLDLVTGVVNIGLT.A | 71.73 | 2439.3262 | 1220.6707 | 0.2 | 2 |
| 204 | K.NIPIVNFDDVLDLVTGVVNIGL.T | 64.10 | 2338.2783 | 1170.1455 | -0.8 | 2 |
| 205 | K.NIPIVNFDDVLDLVTGVVNIGLTA.V | 62.93 | 2510.3633 | 837.7942 | -1.0 | 3 |
| 206 | K.NIPIVNFDDVLDLVTGVVNIGLTAV.G | 57.72 | 2609.4316 | 870.8179 | 0.1 | 3 |
| 207 | K.NIPIVNFDDVLDLVTGVVNIGLTAVG.A | 66.11 | 2666.4531 | 889.8245 | -0.5 | 3 |
| 208 | K.NIPIVNFDDVLDLVTGVVNIGLTAVGAT.A | 78.80 | 2838.5378 | 947.1857 | -0.9 | 3 |
| 209 | K.NIPIVNFDDVLDLVTGVVNIGLTAVGA.T | 62.21 | 2737.4902 | 913.5027 | -1.4 | 3 |
| 210 | K.NIPIVNFDDVLDLVTGVVNIGLTAVGATAG.V | 73.78 | 2966.5964 | 989.8721 | -0.7 | 3 |
| 211 | K.NIPIVNFDDVLDLVTGVVNIGLTAVGATAGVA.I | 71.20 | 3136.7019 | 1046.5756 | 0.9 | 3 |
| 212 | K.NIPIVNFDDVLDLVTGVVNIGLTAVGATA.G | 66.09 | 2909.5750 | 970.8673 | 1.8 | 3 |

Table 6a. List of peptides matches found by nano-HPLC ESI-MS/MS measurements for run 2.

| **Reduced sample L-TYR; run 3 (measured by the Department of Analytical Chemistry)** | |
| --- | --- |
| Device and operating software used | Nano-HPLC (*Dionex Corporation*), Chromeleon Client Version 6.80 (*Dionex Corporation*); LTQ Orbitrap Velos (*Thermo Scientific*), LTQ Tune Plus Version 2.6.0 1065 SP3 (*Thermo Scientific*) |
| Software used (peak list generating) | Xcalibur 2.2 SP1.48 (*Thermo Scientific*) |
| Acquisition parameters | MS1 scan: m/z 400 – 1400; Filling time: 500 ms with 10^6^ ions; Resolution: 60.000; Fragmentation: CID with 35 eV; Peak picking: Top10 (intensity) with isolation window 3 m/z; Resolution; 7.500; Target ion previously selected for fragmentation were dynamically excluded for 180 s with relative mass window of 5 ppm. |
| **Search Parameters** | |
| Search engine | Peaks studio 6.0 |
| Enzyme specify | Trypsin |
| Number of miss cleavages permitted | 3 |
| Number of non-specific cleavage | 0 |
| Fixed modifications (including residue specificity) | 0 |
| Variable modifications (including residue specificity) | Acetylation (*N*-Term) (+42.01 Da), 2-amino-3-oxo-butanoic acid (surrogate for thioether bridge) (-2.02 Da), Val→Ala substitution (-28.03 Da), Ser→Asn substitution (+27.01 Da), methionine oxidation (+15.99 Da), tryptophane oxidation (+15.99 Da) |
| Mass tolerance for precursor ions | ±5 ppm |
| Mass tolerance for fragment ions | ±0.5 Dalton |
| Name of database searched (version/date) | UniProt (UniProtKB/Swiss-Prot, March 2013) |
| Species restriction and justification for searching only a subset of a database | no |
| Number of protein entries in the database actually searched | 35502518 |
| Cut-off score/expectation value for accepting individual MS/MS spectra provided | -10lgP ≥ 15 |
| Software used for PTM determination | Peaks Studio 6.0 |

Table 7a. Experimental settings for nano-HPLC ESI-MS/MS measurements and data evaluation for run 3 with the particular attempt of determining PTMs. Sample: Tryptic digest of a gel band (L-TYR, 62 kDa) from a SDS-PAGE under reducing conditions.

| **Protein identification data (run 3)** | | | |
| --- | --- | --- | --- |
| Accession number | Unique peptides detected | Sequence coverage [%] | Score (-10lgP) |
| C7FF05\|PPO4_AGABI | 79 | 83 | 555.79 |
| P00761\|TRYP_PIG | 5 | 26 | 234.97 |
| P04264\|K2C1_HUMAN | 2 | 14 | 230.42 |
| O42713\|PPO2_AGABI | 2 | 9 | 129.99 |

Table 8a. List of the four polypeptide sequences exhibiting the highest score for matching to the respective MS-data generated by run 3 (nano-HPLC ESI-MS/MS measurements).

| **Identified peptide list (run 3)** | | | | | | |
| --- | --- | --- | --- | --- | --- | --- |
|  | **Sequence** | **-10lgP** | **m (expected)** | **m/z (observed)** | **ppm** | **z** |
| 1 | M.**S(+42.01)**LLATVGPTGGVK.N | 72.24 | 1240.7026 | 621.3574 | -1.9 | 2 |
| 2 | M.SLLATVGPTGGVK.N | 52.03 | 1198.6921 | 600.3525 | -1.3 | 2 |
| 3 | K.NRLDIVDFVR.D | 83.21 | 1245.6830 | 623.8475 | -2.0 | 2 |
| 4 | R.LDIVDFVR.D | 76.22 | 975.5389 | 488.7769 | 0.3 | 2 |
| 5 | R.LDIVDFVRDEK.F | 75.50 | 1347.7034 | 674.8584 | -0.8 | 2 |
| 6 | K.FFTLYVR.A | 70.87 | 944.5120 | 473.2629 | -0.7 | 2 |
| 7 | R.ALQAIQDK.D | 47.02 | 885.4919 | 443.7524 | -1.8 | 2 |
| 8 | K.DQADYSSFFQLSGIHGLPFTPWAKPK.D | 90.42 | 2936.4497 | 735.1176 | -2.8 | 4 |
| 9 | K.DTPTVPYESGYC**T(-2.02)**HSQVLFPTWHR.V | 99.57 | 2818.2810 | 940.4341 | -0.2 | 3 |
| 10 | R.VYVSIYEQVLQEAAK.G | 123.08 | 1738.9141 | 580.6452 | -0.1 | 3 |
| 11 | K.KEW**A(sub V)**QAAEDLR.Q | 88.98 | 1315.6520 | 658.8325 | -1.2 | 2 |
| 12 | K.KEWVQAAEDLR.Q | 76.63 | 1343.6833 | 448.9016 | -0.1 | 3 |
| 13 | K.EW**A(sub V)**QAAEDLR.Q | 90.70 | 1187.5570 | 594.7855 | -0.5 | 2 |
| 14 | K.EW**A(sub V)**QAAEDLRQPYWDTGFALVPPDEIIK.L | 79.34 | 3257.6033 | 815.4068 | -1.6 | 4 |
| 15 | K.EWVQAAEDLR.Q | 23.79 | 1215.5884 | 608.7998 | -2.7 | 2 |
| 16 | R.QPYWDTGFALVPPDEIIK.L | 91.72 | 2088.0566 | 1045.0353 | -0.4 | 2 |
| 17 | K.ITNYDGTK.I | 47.79 | 910.4396 | 456.2277 | 1.5 | 2 |
| 18 | K.ITNYDGTKITVR.N | 35.41 | 1379.7408 | 460.9201 | -1.7 | 3 |
| 19 | R.NPILR.Y | 23.48 | 611.3755 | 612.3835 | 1.2 | 1 |
| 20 | R.YSFHPIDPSFN**(sub S)**GYPNFDTWR.T | 124.03 | 2459.0969 | 1230.5555 | -0.2 | 2 |
| 21 | R.YSFHPIDPSFSGYPNFDTWR.T | 44.97 | 2432.0862 | 1217.0459 | -3.7 | 2 |
| 22 | R.NPDADKKENIPALIAK.L | 60.50 | 1735.9468 | 434.9930 | -2.2 | 4 |
| 23 | K.KENIPALIAK.L | 52.34 | 1095.6652 | 548.8384 | -2.6 | 2 |
| 24 | K.ENIPALIAK.L | 51.16 | 967.5702 | 484.7921 | -0.4 | 2 |
| 25 | K.LDLEADSTR.E | 74.20 | 1018.4930 | 510.2535 | -0.5 | 2 |
| 26 | R.EKTYNMLK.F | 35.88 | 1025.5216 | 513.7672 | -1.7 | 2 |
| 27 | K.TYNMLK.F | 23.40 | 768.3840 | 769.3898 | -1.9 | 1 |
| 28 | K.FNANWEAFSNHGEFDDTHANSLEAVHDDIHGFVGR.G | 78.77 | 3954.7371 | 989.6899 | -1.6 | 4 |
| 29 | R.GHMTHALFAAFDPIFWLHHSNVDR.H | 108.56 | 2818.3550 | 564.6787 | 0.8 | 5 |
| 30 | R.HLSLWQALYPGVWVTQGPER.E | 119.27 | 2336.2065 | 779.7427 | -0.1 | 3 |
| 31 | R.EGSM**(+15.99)**GFAPGTELNK.D | 109.42 | 1452.6555 | 727.3334 | -2.2 | 2 |
| 32 | R.EGSMGFAPGTELNK.D | 87.83 | 1436.6605 | 719.3362 | -1.9 | 2 |
| 33 | R.EGSMGFAPGTELNKDSALEPFYETEDKPWTSVPLTDTALLNYSYPDFDK.V | 48.97 | 5485.5386 | 1098.1168 | 1.7 | 5 |
| 34 | K.DSALEPFYETEDKPWTSVPLTDTALLNYSYPDFDKVK.G | 67.68 | 4294.0522 | 1074.5210 | 0.6 | 4 |
| 35 | K.DSALEPFYETEDKPWTSVPLTDTALLNYSYPDFDK.V | 65.53 | 4066.8887 | 1017.7263 | -3.1 | 4 |
| 36 | K.GGTPDLVR.D | 42.75 | 813.4344 | 814.4399 | -2.2 | 1 |
| 37 | R.DYINDHIDR.R | 72.13 | 1159.5258 | 580.7695 | -1.1 | 2 |
| 38 | K.SEGGKNPALDLLSDFK.G | 120.53 | 1689.8573 | 845.9344 | -1.8 | 2 |
| 39 | K.NPALDLLSDFK.G | 109.55 | 1231.6448 | 616.8287 | -1.5 | 2 |
| 40 | K.GVTHDHNEDLK.M | 45.30 | 1263.5844 | 632.8015 | 3.3 | 2 |
| 41 | K.M**(+15.99)**FDWTIQASWK.K | 140.60 | 1427.6544 | 714.8337 | -1.1 | 2 |
| 42 | K.M**(+15.99)**FDW**(+15.99)**TIQASWK.K | 110.59 | 1443.6493 | 722.8304 | -2.1 | 2 |
| 43 | K.MFDWTIQASWK.K | 95.70 | 1411.6594 | 706.8371 | 0.2 | 2 |
| 44 | K.KFELDDSFAIIFYFAADGSTNVTK.E | 93.45 | 2698.3167 | 900.4465 | 0.4 | 3 |
| 45 | K.ENYIGSINIFR.G | 85.62 | 1324.6775 | 663.3454 | -0.9 | 2 |
| 46 | R.TQDNLVQEGFVHLDR.F | 119.35 | 1769.8696 | 885.9407 | -1.6 | 2 |
| 47 | R.DLDTFDPQAVHR.Y | 99.75 | 1412.6685 | 707.3412 | -0.4 | 2 |
| 48 | K.VVADDHSVTLK.S | 74.56 | 1182.6244 | 592.3190 | -0.8 | 2 |
| 49 | K.VVADDHSVTLKSLR.I | 47.91 | 1538.8417 | 513.9543 | -0.2 | 3 |
| 50 | R.VQGRPLHLPPGVSFPR.L | 33.17 | 1755.9896 | 440.0044 | -0.6 | 4 |

Table 9a. List of peptides matches found by nano-HPLC ESI-MS/MS measurements for run 3.

| **Non-reduced sample L-TYR; run 4 (measured by the Department of Analytical Chemistry)** | |
| --- | --- |
| Device and operating software used | Nano-HPLC (*Dionex Corporation*), Chromeleon Client Version 6.80 (*Dionex Corporation*); LTQ Orbitrap Velos (*Thermo Scientific*), LTQ Tune Plus Version 2.6.0 1065 SP3 (*Thermo Scientific*) |
| Software used (peak list generating) | Xcalibur 2.2 SP1.48 (*Thermo Scientific*) |
| Acquisition parameters | MS1 scan: m/z 400 – 1400; Filling time: 500 ms with 10^6^ ions; Resolution: 60.000; Fragmentation: CID with 35 eV; Peak picking: Top10 (intensity) with isolation window 3 m/z; Resolution; 7.500; Target ion previously selected for fragmentation were dynamically excluded for 180 s with relative mass window of 5 ppm. |
| **Search Parameters** | |
| Search engine | Peaks studio 6.0 |
| Enzyme specify | Trypsin |
| Number of miss cleavages permitted | 3 |
| Number of non-specific cleavage | 1 |
| Fixed modifications (including residue specificity) | 0 |
| Variable modifications (including residue specificity) | Cys-Cys disulfide bridge (-2,02 Da)(searched manually) |
| Mass tolerance for precursor ions | ±5 ppm |
| Mass tolerance for fragment ions | ±0.5 Dalton |
| Name of database searched (version/date) | UniProt (UniProtKB/Swiss-Prot, March 2013) |
| Species restriction and justification for searching only a subset of a database | no |
| Number of protein entries in the database actually searched | 35502518 |
| Cut-off score/expectation value for accepting individual MS/MS spectra provided | -10lgP ≥ 15 |
| Software used for PTM determination | Peaks Studio 6.0 |

Table 10a. Experimental settings for nano-HPLC ESI-MS/MS measurements and data evaluation for run 4. Sample: Tryptic digest of a gel band (L-TYR, 62 kDa) from a SDS-PAGE under non-reducing conditions.

| **Protein identification data (run 4)** | | | |
| --- | --- | --- | --- |
| Accession number | Unique peptides detected | Sequence coverage [%] | Score (-10lgP) |
| C7FF05\|PPO4_AGABI | 135 | 68 | 409.19 |
| P00761\|TRYP_PIG | 12 | 29 | 169.94 |
| P17505\|MDHM_YEAST | 1 | 3 | 54.01 |
| P31301\|PYRC_USTMA | 1 | 2 | 50-45 |
| A2BE76\|CP072_DANRE | 1 | 3 | 42.44 |

Table 11a. List of the five polypeptide sequences exhibiting the highest score for matching to the respective MS-data generated by run 4(nano-HPLC ESI-MS/MS measurements).

| **Identified peptide list (run 4)** | | | | | | |
| --- | --- | --- | --- | --- | --- | --- |
|  | **Sequence** | **-10lgP** | **m (expected)** | **m/z (observed)** | **ppm** | **z** |
| 1 | M.SLLATVGPTGGVK.N | 33.53 | 1198.6921 | 600.3527 | -1.0 | 2 |
| 2 | S.LLATVGPTGGVK.N | 29.65 | 1111.6600 | 556.8369 | -0.8 | 2 |
| 3 | L.LATVGPTGGVK.N | 27.61 | 998.5760 | 500.2955 | 0.5 | 2 |
| 4 | A.TVGPTGGVK.N | 16.53 | 814.4548 | 815.4607 | -1.7 | 1 |
| 5 | K.NRLDIVDFVR.D | 65.87 | 1245.6830 | 623.8480 | -1.3 | 2 |
| 6 | R.LDIVDFVR.D | 55.15 | 975.5389 | 488.7761 | -1.2 | 2 |
| 7 | L.DIVDFVR.D | 35.23 | 862.4548 | 432.2343 | -1.0 | 2 |
| 8 | D.IVDFVR.D | 16.86 | 747.4279 | 748.4330 | -2.9 | 1 |
| 9 | K.FFTLYVR.A | 46.81 | 944.5120 | 473.2628 | -0.8 | 2 |
| 10 | R.ALQAIQDKDQAD.Y | 56.71 | 1314.6415 | 658.3271 | -1.3 | 2 |
| 11 | R.ALQAIQDK.D | 38.00 | 885.4919 | 443.7531 | -0.2 | 2 |
| 12 | K.DQADYSSFFQLSGIHGLPFTPW.A | 42.04 | 2512.1699 | 838.3981 | 1.1 | 3 |
| 13 | L.SGIHGLPFTPWAKPK.D | 45.17 | 1634.8932 | 409.7303 | -0.7 | 4 |
| 14 | H.SQVLFPTWHR.V | 36.03 | 1269.6619 | 424.2274 | -1.1 | 3 |
| 15 | Q.VLFPTWHR.V | 38.48 | 1054.5712 | 528.2920 | -1.6 | 2 |
| 16 | L.FPTWHR.V | 15.46 | 842.4188 | 422.2160 | -1.6 | 2 |
| 17 | Y.EQVLQEAAK.G | 21.09 | 1014.5345 | 508.2747 | 0.2 | 2 |
| 18 | R.QPYWDTGFALVPPDEIIK.L | 48.95 | 2088.0566 | 1045.0363 | 0.6 | 2 |
| 19 | Y.WDTGFALVPPDEIIK.L | 59.08 | 1699.8821 | 850.9476 | -0.8 | 2 |
| 20 | W.DTGFALVPPDEIIK.L | 42.89 | 1513.8027 | 757.9075 | -1.5 | 2 |
| 21 | F.ALVPPDEIIK.L | 21.64 | 1093.6383 | 547.8263 | -0.2 | 2 |
| 22 | L.VPPDEIIK.L | 17.22 | 909.5171 | 455.7640 | -4.0 | 2 |
| 23 | V.PPDEIIK.L | 23.46 | 810.4487 | 811.4550 | -1.2 | 1 |
| 24 | Y.PNFDTWR.T | 31.80 | 934.4297 | 468.2218 | -0.7 | 2 |
| 25 | P.NFDTWR.T | 24.12 | 837.3770 | 419.6953 | -0.9 | 2 |
| 26 | R.NPDADKKENIPALIAK.L | 57.79 | 1735.9468 | 579.6547 | -2.5 | 3 |
| 27 | K.KENIPALIAK.L | 44.90 | 1095.6652 | 548.8384 | -2.6 | 2 |
| 28 | K.ENIPALIAK.L | 32.04 | 967.5702 | 968.5757 | -1.8 | 1 |
| 29 | K.LDLEADSTR.E | 55.38 | 1018.4930 | 510.2531 | -1.3 | 2 |
| 30 | L.DLEADSTR.E | 21.49 | 905.4090 | 906.4124 | -4.2 | 1 |
| 31 | D.LEADSTR.E | 18.60 | 790.3821 | 791.3860 | -4.2 | 1 |
| 32 | K.FNANWEAFSNHGEFDDTHANSLEAVHDDIHGFVGR.G | 73.00 | 3954.7371 | 989.6899 | -1.6 | 4 |
| 33 | K.FNANWEAFSNHGEFDDTHANSL.E | 69.51 | 2522.0522 | 841.6901 | -1.5 | 3 |
| 34 | K.FNANWEAFSNHGEFDDTHAN.S | 58.94 | 2321.9363 | 774.9844 | -2.0 | 3 |
| 35 | K.FNANWEAFSNHGEFDDTHANSLEAVHDDIHGF.V | 57.99 | 3642.5461 | 911.6419 | -2.1 | 4 |
| 36 | K.FNANWEAFSN.H | 51.32 | 1198.5043 | 600.2593 | -0.1 | 2 |
| 37 | K.FNANWEAFSNH.G | 35.91 | 1335.5632 | 668.7880 | -1.3 | 2 |
| 38 | K.FNANWEAF.S | 32.47 | 997.4293 | 499.7216 | -0.7 | 2 |
| 39 | K.FNANWEA.F | 22.87 | 850.3610 | 426.1872 | -1.3 | 2 |
| 40 | F.NANWEAFSNHGEFDDTHANSLEAVHDDIHGFVGR.G | 59.31 | 3807.6687 | 952.9235 | -1.0 | 4 |
| 41 | N.ANWEAFSNHGEFDDTHANSLEAVHDDIHGFVGR.G | 58.03 | 3693.6257 | 739.7319 | -0.7 | 5 |
| 42 | N.WEAFSNHGEFDDTHANSLEAVHDDIHGFVGR.G | 72.01 | 3508.5457 | 878.1423 | -1.5 | 4 |
| 43 | F.SNHGEFDDTHANSLEAVHDDIHGFVGR.G | 87.08 | 2975.3181 | 744.8361 | -0.9 | 4 |
| 44 | N.HGEFDDTHANSLEAVHDDIHGFVGR.G | 91.96 | 2774.2432 | 694.5672 | -1.2 | 4 |
| 45 | N.SLEAVHDDIHGFVGR.G | 85.06 | 1650.8114 | 826.4117 | -1.6 | 2 |
| 46 | L.EAVHDDIHGFVGR.G | 63.34 | 1450.6953 | 726.3516 | -4.6 | 2 |
| 47 | A.VHDDIHGFVGR.G | 38.01 | 1250.6156 | 417.8792 | 0.1 | 3 |
| 48 | R.GHMTHALFAAFDPIFWLHHSNVDR.H | 95.37 | 2818.3550 | 564.6774 | -1.6 | 5 |
| 49 | R.GHMTHALF.A | 44.31 | 912.4276 | 913.4343 | -0.7 | 1 |
| 50 | R.GHMTHALFAAFDPIFWLH.H | 42.34 | 2110.0247 | 528.5132 | -0.5 | 4 |
| 51 | R.GHMTHALFA.A | 29.23 | 983.4647 | 492.7390 | -1.3 | 2 |
| 52 | H.MTHALFAAFDPIFWLHHSNVDR.H | 66.48 | 2624.2747 | 525.8617 | -0.9 | 5 |
| 53 | H.ALFAAFDPIFWLHHSNVDR.H | 78.65 | 2255.1274 | 1128.5691 | -1.7 | 2 |
| 54 | A.LFAAFDPIFWLHHSNVDR.H | 55.89 | 2184.0903 | 547.0302 | 0.7 | 4 |
| 55 | L.FAAFDPIFWLHHSNVDR.H | 69.93 | 2071.0063 | 691.3423 | -0.5 | 3 |
| 56 | F.AAFDPIFWLHHSNVDR.H | 72.64 | 1923.9380 | 642.3192 | -1.2 | 3 |
| 57 | A.AFDPIFWLHHSNVDR.H | 52.73 | 1852.9009 | 618.6396 | -2.1 | 3 |
| 58 | A.FDPIFWLHHSNVDR.H | 53.14 | 1781.8638 | 446.4726 | -1.4 | 4 |
| 59 | R.HLSLWQALYPGVWVTQGPER.E | 83.83 | 2336.2065 | 779.7418 | -1.3 | 3 |
| 60 | R.HLSLWQAL.Y | 27.70 | 966.5287 | 967.5342 | -1.8 | 1 |
| 61 | R.HLSLWQA.L | 19.38 | 853.4446 | 427.7294 | -0.5 | 2 |
| 62 | R.HLSLWQ.A | 15.56 | 782.4075 | 783.4141 | -0.9 | 1 |
| 63 | R.HLSLW.Q | 15.24 | 654.3489 | 655.3553 | -1.4 | 1 |
| 64 | Y.PGVWVTQGPER.E | 61.06 | 1224.6251 | 613.3185 | -2.2 | 2 |
| 65 | P.GVWVTQGPER.E | 48.75 | 1127.5724 | 564.7925 | -1.7 | 2 |
| 66 | R.EGSMGFAPGTELNK.D | 75.28 | 1436.6605 | 719.3358 | -2.5 | 2 |
| 67 | R.EGSMGFAPGTEL.N | 40.55 | 1194.5227 | 1195.5276 | -2.0 | 1 |
| 68 | R.EGSMGFAPGTELNKDSALEPFYETEDKPWTSVPLTDTALLNYSYPDFDK.V | 35.42 | 5485.5386 | 1372.3894 | -1.9 | 4 |
| 69 | E.GSMGFAPGTELNK.D | 60.52 | 1307.6179 | 654.8154 | -1.2 | 2 |
| 70 | G.SMGFAPGTELNK.D | 51.27 | 1250.5964 | 626.3044 | -1.8 | 2 |
| 71 | S.MGFAPGTELNK.D | 53.01 | 1163.5645 | 582.7892 | -0.5 | 2 |
| 72 | K.DSALEPFYETEDKPWTSVPLTDTALLNYSYPDFDK.V | 65.30 | 4066.8887 | 1017.7324 | 2.9 | 4 |
| 73 | K.DSALEPFYETEDKPWTSVPLTDTALLNYSYPDFD.K | 24.17 | 3938.7937 | 1313.9362 | -1.9 | 3 |
| 74 | K.GGTPDLVR.D | 38.66 | 813.4344 | 814.4409 | -1.0 | 1 |
| 75 | K.GGTPDLVRD.Y | 25.11 | 928.4614 | 465.2382 | 0.6 | 2 |
| 76 | K.SEGGKNPALDLLSDFK.G | 55.04 | 1689.8573 | 564.2916 | -2.5 | 3 |
| 77 | K.NPALDLLSDFK.G | 75.89 | 1231.6448 | 616.8289 | -1.2 | 2 |
| 78 | K.NPALDLLSD.F | 24.56 | 956.4814 | 479.2475 | -1.0 | 2 |
| 79 | K.NPALDLLS.D | 23.80 | 841.4545 | 842.4604 | -1.7 | 1 |
| 80 | N.PALDLLSDFK.G | 64.52 | 1117.6019 | 559.8079 | -0.7 | 2 |
| 81 | P.ALDLLSDFK.G | 42.86 | 1020.5491 | 511.2810 | -1.6 | 2 |
| 82 | K.MFDWTIQASWK.K | 84.94 | 1411.6594 | 706.8361 | -1.3 | 2 |
| 83 | K.MFDWTIQAS.W | 45.80 | 1097.4852 | 549.7492 | -1.2 | 2 |
| 84 | K.MFDWTIQASW.K | 36.02 | 1283.5645 | 642.7893 | -0.3 | 2 |
| 85 | K.MFDWTIQA.S | 28.80 | 1010.4531 | 506.2334 | -0.9 | 2 |
| 86 | K.MFDWTIQ.A | 19.97 | 939.4160 | 470.7150 | -0.5 | 2 |
| 87 | M.FDWTIQASWK.K | 61.71 | 1280.6189 | 641.3149 | -2.8 | 2 |
| 88 | K.KFELDDSFAIIFYFAADGSTNVTK.E | 81.35 | 2698.3167 | 900.4451 | -1.2 | 3 |
| 89 | K.KFELDDSFAIIFYF.A | 47.71 | 1753.8602 | 877.9368 | -0.7 | 2 |
| 90 | K.KFELDDSFAIIFY.F | 39.67 | 1606.7919 | 804.4034 | 0.3 | 2 |
| 91 | K.KFELDDSFAIIF.Y | 38.37 | 1443.7285 | 722.8712 | -0.5 | 2 |
| 92 | K.KFELDDSF.A | 30.48 | 999.4549 | 500.7343 | -0.7 | 2 |
| 93 | K.FELDDSFAIIFYFAADGSTNVTK.E | 87.47 | 2570.2217 | 857.7476 | -0.2 | 3 |
| 94 | Y.FAADGSTNVTK.E | 46.65 | 1109.5353 | 555.7750 | 0.2 | 2 |
| 95 | Y.IGSINIFR.G | 25.47 | 918.5287 | 460.2702 | -3.1 | 2 |
| 96 | R.GTTPTN(**C**AN**C**-2.02)R.T * | x | 1137.2661 | 568.7215 | 25 | 2 |
| 97 | C.RTQDNLVQEGFVHLDR.F | 47.14 | 1925.9707 | 642.9946 | -4.5 | 3 |
| 98 | R.TQDNLVQEGFVHLDR.F | 91.04 | 1769.8696 | 885.9398 | -2.6 | 2 |
| 99 | R.TQDNLVQEGFVHLD.R | 76.47 | 1613.7686 | 807.8905 | -1.3 | 2 |
| 100 | R.TQDNLVQEGFVH.L | 61.64 | 1385.6575 | 693.8348 | -1.8 | 2 |
| 101 | R.TQDNLVQEGFVHL.D | 56.30 | 1498.7416 | 750.3758 | -3.0 | 2 |
| 102 | R.TQDNLVQEGF.V | 44.55 | 1149.5302 | 1150.5360 | -1.3 | 1 |
| 103 | T.QDNLVQEGFVHLDR.F | 65.94 | 1668.8219 | 835.4160 | -2.6 | 2 |
| 104 | Q.DNLVQEGFVHLDR.F | 34.89 | 1540.7634 | 514.5943 | -1.5 | 3 |
| 105 | N.LVQEGFVHLDR.F | 56.71 | 1311.6935 | 656.8533 | -1.1 | 2 |
| 106 | R.DLDTFDPQAVHR.Y | 78.26 | 1412.6685 | 707.3400 | -2.2 | 2 |
| 107 | R.DLDTFDPQAVH.R | 58.44 | 1256.5673 | 629.2897 | -1.9 | 2 |
| 108 | R.DLDTFDPQ.A | 28.66 | 949.4028 | 475.7084 | -0.5 | 2 |
| 109 | D.LDTFDPQAVHR.Y | 36.92 | 1297.6415 | 433.5540 | -1.1 | 3 |
| 110 | L.DTFDPQAVHR.Y | 51.21 | 1184.5574 | 593.2848 | -2.0 | 2 |
| 111 | D.TFDPQAVHR.Y | 29.69 | 1069.5305 | 535.7707 | -3.4 | 2 |
| 112 | T.FDPQAVHR.Y | 29.36 | 968.4828 | 485.2464 | -4.6 | 2 |
| 113 | Y.KVVADDHSVTLK.S | 28.25 | 1310.7194 | 437.9138 | 0.2 | 3 |
| 114 | K.VVADDHSVTLK.S | 66.82 | 1182.6244 | 592.3188 | -1.1 | 2 |
| 115 | K.VVADDHSVTL.K | 34.75 | 1054.5294 | 528.2717 | -0.5 | 2 |
| 116 | V.VADDHSVTLK.S | 43.63 | 1083.5560 | 542.7852 | -0.1 | 2 |
| 117 | V.ADDHSVTLK.S | 18.62 | 984.4876 | 493.2511 | 0.1 | 2 |
| 118 | D.DHSVTLK.S | 19.13 | 798.4236 | 400.2171 | -4.9 | 2 |
| 119 | R.PLHLPPGVSFPR.L | 58.38 | 1315.7400 | 439.5869 | -0.9 | 3 |
| 120 | P.LHLPPGVSFPR.L | 48.51 | 1218.6873 | 407.2359 | -1.0 | 3 |
| 121 | L.PPGVSFPR.L | 31.10 | 855.4603 | 428.7361 | -2.9 | 2 |
| 122 | K.NIPIVNFDDVLDLVTGVVNIG.L | 71.91 | 2225.1943 | 742.7380 | -1.0 | 3 |
| 123 | K.NIPIVNFDDVLDLVTGVVNIGLTAV.G | 66.46 | 2609.4316 | 870.8170 | -1.0 | 3 |
| 124 | K.NIPIVNFDDVLDLVTGVVNIGLTAVGAT.A | 66.40 | 2838.5378 | 947.1820 | -4.8 | 3 |
| 125 | K.NIPIVNFDDVLDLVTGVVNIGLTAVGATA.G | 62.04 | 2909.5750 | 970.8639 | -1.8 | 3 |
| 126 | K.NIPIVNFDDVLDLVTGVVN.I | 61.03 | 2055.0889 | 686.0367 | -0.3 | 3 |
| 127 | K.NIPIVNFDDVLDLVTGVVNIGLTAVG.A | 59.60 | 2666.4531 | 889.8243 | -0.8 | 3 |
| 128 | K.NIPIVNFDDVLDLVTGVVNIGLTA.V | 59.37 | 2510.3633 | 837.7932 | -2.2 | 3 |
| 129 | K.NIPIVNFDDVLDLVTGVVNIGLT.A | 59.35 | 2439.3262 | 1220.6711 | 0.6 | 2 |
| 130 | K.NIPIVNFDDVLDLVTGVVNIGL.T | 58.27 | 2338.2783 | 1170.1438 | -2.3 | 2 |
| 131 | K.NIPIVNFDDVLDLVT.G | 57.73 | 1685.8876 | 843.9513 | 0.3 | 2 |
| 132 | K.NIPIVNFDDVLDLVTGVVNIGLTAVGA.T | 56.54 | 2737.4902 | 913.5024 | -1.8 | 3 |
| 133 | K.NIPIVNFDDVLDLVTGVV.N | 55.34 | 1941.0459 | 971.5292 | -1.1 | 2 |
| 134 | K.NIPIVNFDDVLDLVTGV.V | 52.17 | 1841.9774 | 921.9948 | -1.3 | 2 |
| 135 | K.NIPIVNFDDVLDLVTGVVNIGLTAVGATAG.V | 51.79 | 2966.5964 | 989.8721 | -0.7 | 3 |
| 136 | K.NIPIVNFDDVLDLVTGVVNIGLTAVGATAGVA.I | 42.63 | 3136.7019 | 1046.5743 | -0.2 | 3 |

Table 12a. List of peptides matches found by nano-HPLC ESI-MS/MS measurements for run 4.

| **Reduced sample L-TYR vs. K9I869; run 5 (measured by the Department of Analytical Chemistry)** | |
| --- | --- |
| Device and operating software used | Nano-HPLC (*Dionex Corporation*), Chromeleon Client Version 6.80 (*Dionex Corporation*); LTQ Orbitrap Velos (*Thermo Scientific*), LTQ Tune Plus Version 2.6.0 1065 SP3 (*Thermo Scientific*) |
| Software used (peak list generating) | Xcalibur 2.2 SP1.48 (*Thermo Scientific*) |
| Acquisition parameters | MS1 scan: m/z 400 – 1400; Filling time: 500 ms with 10^6^ ions; Resolution: 60.000; Fragmentation: CID with 35 eV; Peak picking: Top10 (intensity) with isolation window 3 m/z; Resolution; 7.500; Target ion previously selected for fragmentation were dynamically excluded for 180 s with relative mass window of 5 ppm. |
| **Search Parameters** | |
| Search engine | Peaks studio 6.0 |
| Enzyme specify | Trypsin |
| Number of miss cleavages permitted | 3 |
| Number of non-specific cleavage | 1 |
| Fixed modifications (including residue specificity) | 0 |
| Variable modifications (including residue specificity) | Ala->Val substitution (+28.03 Da), Gly->Ala substitution (+14.02 Da),  Ile->Val substitution (-14.02 Da), Lys->Arg substitution (+28.01 Da), Leu->Val substitution (-14.02 Da), Asn->Ser substitution (-27.01 Da), Gln->Leu substitution ( -14.97 Da), Ser->Ala substitution (-15.99 Da) |
| Mass tolerance for precursor ions | ±5 ppm |
| Mass tolerance for fragment ions | ±0.5 Dalton |
| Name of database searched (version/date) | UniProt. Entry \|K9I869\|K9I869_AGABB |
| Cut-off score/expectation value for accepting individual MS/MS spectra provided | -10lgP ≥ 15 |
| Software used for PTM determination | Peaks Studio 6.0 |

Table 13a. Experimental settings for nano-HPLC ESI-MS/MS measurements and data evaluation for run 5. Sample: Tryptic digest of a gel band (L-TYR, 62 kDa) from a SDS-PAGE under reducing conditions vs. the single sequence of UniProt entry |K9I869|K9I869_AGABB.

| **Protein identification data (run 5)** | | | |
| --- | --- | --- | --- |
| Accession number | Unique peptides detected | Sequence coverage [%] | Score (-10lgP) |
| \|K9I869\|K9I869_AGABB | 345 | 87 | 538.17 |

Table 14a. Polypeptide sequences exhibiting the highest score for matching to the respective MS-data generated by run 5 (nano-HPLC ESI-MS/MS measurements).

| **Identified peptide list (run 5)** | | | | | | |
| --- | --- | --- | --- | --- | --- | --- |
|  | **Sequence** | **-10lgP** | **m (expected)** | **m/z (observed)** | **ppm** | **z** |
| 1 | M.SLLATVGPTGGVK.N | 56.8 | 1198.6921 | 600.3524 | -2 | 2 |
| 2 | S.LLATVGPTGGVK.N | 43.4 | 1111.66 | 556.8363 | -2 | 2 |
| 3 | L.LATVGPTGGVK.N | 42.7 | 998.576 | 500.2944 | -2 | 2 |
| 4 | L.ATVGPTGGVK.N | 42.9 | 885.4919 | 443.7546 | 3 | 2 |
| 5 | K.NRLDIVDFVR.D | 68.7 | 1245.683 | 623.8475 | -2 | 2 |
| 6 | N.RLDIVDFVR.D | 51.0 | 1131.64 | 566.827 | -1 | 2 |
| 7 | R.LDIVDFVRDEK.F | 70.2 | 1347.7034 | 674.8584 | -1 | 2 |
| 8 | R.LDIVDFVR.D | 67.7 | 975.5389 | 488.7769 | 0 | 2 |
| 9 | L.DIVDFVR.D | 39.7 | 862.4548 | 863.4602 | -2 | 1 |
| 10 | D.IVDFVR.D | 25.1 | 747.4279 | 748.4336 | -2 | 1 |
| 11 | I.VDFVR.D | 19.3 | 634.3438 | 635.3498 | -2 | 1 |
| 12 | K.FFTLYIR.A | 68.7 | 958.5276 | 480.2706 | -1 | 2 |
| 13 | K.FFTLYV**(sub L)**R.A | 63.4 | 944.512 | 473.2629 | -1 | 2 |
| 14 | K.FFTLY.I | 20.3 | 689.3424 | 690.3484 | -2 | 1 |
| 15 | R.ALQAIQDKDQSDYSSFFQLSGIHGLPFTPWAK.P | 94.9 | 3594.7783 | 899.7007 | -1 | 4 |
| 16 | R.ALQAIQDKDQA**(sub S)**DYSSFFQLSGIHGLPFTPWAK.P | 90.4 | 3578.7832 | 895.7042 | 1 | 4 |
| 17 | R.ALQAIQDKDQSDYSSFFQL.S | 75.6 | 2203.0432 | 1102.5276 | -1 | 2 |
| 18 | R.ALQAIQDKDQSDYSSFFQLSGIHGLPFTPW.A | 69.3 | 3395.6462 | 849.9187 | 0 | 4 |
| 19 | R.ALQAIQDKDQA**(sub S)**DYSSFFQL.S | 66.4 | 2187.0483 | 730.0224 | -1 | 3 |
| 20 | R.ALQAIQDKDQA**(sub S)**DYSSFFQLSGIHGLPFTPW.A | 65.1 | 3379.6514 | 845.9191 | -1 | 4 |
| 21 | R.ALQAIQDKDQSD.Y | 62.7 | 1330.6365 | 666.324 | -2 | 2 |
| 22 | R.ALQAIQDKDQA**(sub S)**DYSSFFQLSGIHGLPF.T | 60.3 | 2995.4714 | 999.4998 | 2 | 3 |
| 23 | R.ALQAIQDKDQSDYSSFFQLSGIHGLPF.T | 60.1 | 3011.4663 | 1004.8294 | 0 | 3 |
| 24 | R.ALQAIQDK.D | 56.1 | 885.4919 | 886.4982 | -1 | 1 |
| 25 | R.ALQAIQDKD.Q | 46.9 | 1000.5189 | 501.2663 | -1 | 2 |
| 26 | R.ALQAIQD.K | 31.2 | 757.397 | 758.4032 | -1 | 1 |
| 27 | A.LQAIQDK.D | 33.5 | 814.4548 | 408.2337 | -2 | 2 |
| 28 | K.DQSDYSSFFQLSGIHGLPFTPWAK.P | 95.4 | 2727.2969 | 682.831 | -1 | 4 |
| 29 | K.DQA**(sub S)**DYSSFFQLSGIHGLPFTPWAK.P | 93.9 | 2711.302 | 904.7749 | 0 | 3 |
| 30 | K.DQA**(sub S)**DYSSFFQLSGIHGLPFTPWAKPK.D | 84.6 | 2936.4497 | 735.1176 | -3 | 4 |
| 31 | K.DQSDYSSFFQLSGIHGLPFTPWAKPK.D | 84.1 | 2952.4446 | 739.1168 | -2 | 4 |
| 32 | K.DQA**(sub S)**DYSSFFQLSGIHGLPFTPW.A | 81.2 | 2512.1699 | 838.3985 | 2 | 3 |
| 33 | K.DQA**(sub S)**DYSSFFQLSGIH.G | 79.0 | 1713.7634 | 857.8887 | 0 | 2 |
| 34 | K.DQSDYSSFFQLSGIH.G | 76.5 | 1729.7583 | 865.8861 | 0 | 2 |
| 35 | K.DQA**(sub S)**DYSSFFQLSGIHGLPF.T | 76.3 | 2127.99 | 1065.0031 | 1 | 2 |
| 36 | K.DQSDYSSFFQLSGIHGLPF.T | 75.6 | 2143.9851 | 1073.0001 | 0 | 2 |
| 37 | K.DQSDYSSFFQLSGIHGLPFTPW.A | 75.5 | 2528.1648 | 1265.0913 | 1 | 2 |
| 38 | K.DQSDYSSFFQL.S | 65.4 | 1335.5619 | 668.788 | 0 | 2 |
| 39 | K.DQA**(sub S)**DYSSFFQL.S | 63.5 | 1319.567 | 660.7911 | 1 | 2 |
| 40 | K.DQA**(sub S)**DYSSFFQLS.G | 54.3 | 1406.599 | 704.308 | 2 | 2 |
| 41 | K.DQSDYSSFFQLS.G | 51.9 | 1422.5939 | 712.3049 | 1 | 2 |
| 42 | K.DTPTVPYESGY.C | 48.5 | 1227.5295 | 1228.5359 | -1 | 1 |
| 43 | K.DTPTVPY.E | 41.0 | 791.3701 | 792.3765 | -1 | 1 |
| 44 | K.DTPTVPYESGYCTHSQVLFPTWHR.V | 17.3 | 2820.2966 | 941.1016 | -5 | 3 |
| 45 | D.TPTVPYESGYCTHSQVLFPTWHR.V | 29.3 | 2705.2695 | 677.3215 | -5 | 4 |
| 46 | H.SQVLFPTWHR.V | 62.9 | 1269.6619 | 635.8378 | -1 | 2 |
| 47 | Q.VLFPTWHR.V | 51.4 | 1054.5712 | 528.2926 | -1 | 2 |
| 48 | V.LFPTWHR.V | 15.8 | 955.5028 | 478.7581 | -1 | 2 |
| 49 | L.FPTWHR.V | 37.6 | 842.4188 | 422.2164 | -1 | 2 |
| 50 | R.VYVSIYEQILQEAAK.G | 104.1 | 1752.9297 | 585.3168 | -1 | 3 |
| 51 | R.VYVSIYEQV**(sub L)**LQEAAK.G | 95.6 | 1738.9141 | 580.6452 | 0 | 3 |
| 52 | R.VYVSIYEQV**(sub L)**LQEV(sub A)AK.G | 90.5 | 1766.9454 | 884.4796 | 0 | 2 |
| 53 | R.VYVSIYEQV**(sub L)**LQEA.A | 76.7 | 1539.782 | 770.8968 | -2 | 2 |
| 54 | R.VYVSIYEQILQEA.A | 76.2 | 1553.7977 | 777.9069 | 1 | 2 |
| 55 | R.VYVSIYEQ.I | 53.5 | 999.4913 | 500.7525 | -1 | 2 |
| 56 | R.VYVSIY.E | 26.9 | 742.3901 | 743.397 | -1 | 1 |
| 57 | Y.VSIYEQILQEAAK.G | 85.6 | 1490.798 | 746.4065 | 0 | 2 |
| 58 | Y.VSIYEQV**(sub L)**LQEAAK.G | 73.9 | 1476.7823 | 739.3976 | -1 | 2 |
| 59 | S.IYEQILQEAAK.G | 72.0 | 1304.6975 | 653.355 | -2 | 2 |
| 60 | Y.EQILQEAAK.G | 42.8 | 1028.5502 | 515.2817 | -1 | 2 |
| 61 | K.KEWAQAAEDLR.Q | 76.5 | 1315.652 | 658.8329 | -1 | 2 |
| 62 | K.KEWV**(sub A)**QAAEDLR.Q | 64.5 | 1343.6833 | 448.9016 | 0 | 3 |
| 63 | K.EWAQAAEDLRQPY.W | 77.2 | 1575.7317 | 526.2502 | -2 | 3 |
| 64 | K.EWAQAAEDLR.Q | 68.6 | 1187.557 | 594.7855 | -1 | 2 |
| 65 | K.EWAQAAEDLRQPYWDTGFALVPPDEIIK.L | 66.5 | 3257.6033 | 815.4068 | -2 | 4 |
| 66 | E.WAQAAEDLR.Q | 51.8 | 1058.5145 | 530.2643 | 0 | 2 |
| 67 | R.QPYWDTGFALVPPDEIIK.L | 71.5 | 2088.0566 | 1045.0349 | -1 | 2 |
| 68 | R.QPYWDTGF.A | 37.7 | 1012.429 | 507.2216 | 0 | 2 |
| 69 | R.QPYWDTGFALVPPD.E | 31.3 | 1604.7511 | 803.3833 | 1 | 2 |
| 70 | Q.PYWDTGFALVPPDEIIK.L | 81.9 | 1959.9982 | 981.0062 | 0 | 2 |
| 71 | P.YWDTGFALVPPDEIIK.L | 78.3 | 1862.9454 | 932.4794 | -1 | 2 |
| 72 | Y.WDTGFALVPPDEIIK.L | 68.8 | 1699.8821 | 850.9488 | 1 | 2 |
| 73 | D.TGFALVPPDEIIK.L | 50.2 | 1398.7758 | 700.394 | -2 | 2 |
| 74 | G.FALVPPDEIIK.L | 44.0 | 1240.7067 | 621.3603 | -1 | 2 |
| 75 | F.ALVPPDEIIK.L | 34.3 | 1093.6383 | 547.8254 | -2 | 2 |
| 76 | V.PPDEIIK.L | 34.1 | 810.4487 | 811.4548 | -1 | 1 |
| 77 | K.ITNYDGTK.I | 56.9 | 910.4396 | 456.2277 | 2 | 2 |
| 78 | K.ITNYDGTKITVR.N | 41.2 | 1379.7408 | 460.9201 | -2 | 3 |
| 79 | R.NPILR.Y | 27.8 | 611.3755 | 612.3835 | 1 | 1 |
| 80 | R.YSFHPIDPSFNGYPNFDTWK.T | 98.8 | 2431.0908 | 1216.5529 | 0 | 2 |
| 81 | R.YSFHPIDPSFNGYPNFDTWR**(sub K)**.T | 92.1 | 2459.0969 | 1230.5555 | 0 | 2 |
| 82 | R.YSFHPIDPSFNGYPNFD.T | 69.1 | 2015.8689 | 1008.9398 | -2 | 2 |
| 83 | R.YSFHPIDPSFNGYPN.F | 64.0 | 1753.7736 | 877.8945 | 1 | 2 |
| 84 | R.YSFHPIDPSFN.G | 61.5 | 1322.5931 | 662.3036 | 0 | 2 |
| 85 | R.YSFHPIDPSF.N | 50.6 | 1208.5502 | 605.2822 | 0 | 2 |
| 86 | R.YSFHPID.P | 44.7 | 877.397 | 439.7052 | -1 | 2 |
| 87 | R.YSFHPIDPSFS**(sub N)**GYPNFDTWR**(sub K)**.T | 43.8 | 2432.0862 | 811.6995 | -4 | 3 |
| 88 | R.YSFHPIDPS.F | 17.5 | 1061.4818 | 531.7484 | 0 | 2 |
| 89 | D.PSFNGYPNFDTWK.T | 77.6 | 1571.7045 | 786.8593 | 0 | 2 |
| 90 | D.PSFNGYPNFDTWR**(sub K)**.T | 71.2 | 1599.7106 | 800.8613 | -2 | 2 |
| 91 | S.FNGYPNFDTWK.T | 43.0 | 1387.6196 | 694.8163 | -1 | 2 |
| 92 | N.GYPNFDTWR**(sub K)**.T | 49.1 | 1154.5145 | 578.2642 | -1 | 2 |
| 93 | N.FDTWK.T | 21.4 | 695.3279 | 696.3348 | 0 | 1 |
| 94 | R.NPDADKKENIPALIGK.L | 95.9 | 1721.9312 | 861.9719 | -1 | 2 |
| 95 | R.NPDADKKENIPALIA**(sub G)**K.L | 61.1 | 1735.9468 | 434.9937 | -1 | 4 |
| 96 | K.KENIPALIGK.L | 58.9 | 1081.6495 | 1082.6553 | -2 | 1 |
| 97 | K.KENIPALIA**(sub G)**K.L | 54.0 | 1095.6652 | 548.8384 | -3 | 2 |
| 98 | K.ENIPALIGK.L | 55.5 | 953.5546 | 954.5609 | -1 | 1 |
| 99 | K.ENIPALIA**(sub G)**K.L | 53.8 | 967.5702 | 484.7921 | 0 | 2 |
| 100 | N.IPALIGK.L | 23.1 | 710.469 | 711.4743 | -3 | 1 |
| 101 | N.IPALIA**(sub G)**K.L | 16.4 | 724.4847 | 725.4904 | -2 | 1 |
| 102 | I.PALIGK.L | 15.9 | 597.3849 | 598.3915 | -1 | 1 |
| 103 | G.KLDLEADSTR.E | 56.5 | 1146.588 | 574.2997 | -3 | 2 |
| 104 | K.LDLEADSTR.E | 65.5 | 1018.493 | 1019.4996 | -1 | 1 |
| 105 | K.LDLEAD.S | 21.8 | 674.3123 | 675.3182 | -2 | 1 |
| 106 | L.DLEADSTR.E | 39.2 | 905.409 | 906.4149 | -2 | 1 |
| 107 | D.LEADSTR.E | 40.7 | 790.3821 | 791.3883 | -1 | 1 |
| 108 | L.EADSTR.E | 25.7 | 677.298 | 678.3039 | -2 | 1 |
| 109 | R.EKTYNMLK.F | 45.0 | 1025.5216 | 513.7672 | -2 | 2 |
| 110 | K.TYNMLK.F | 32.5 | 768.384 | 769.3907 | -1 | 1 |
| 111 | K.FNANWEAFSNHGEFDDTHANSLEAVHDDIHGFVGR.G | 89.4 | 3954.7371 | 989.6899 | -2 | 4 |
| 112 | K.FNANWEAFSNHGEFDDTHANSL.E | 76.8 | 2522.0522 | 841.6911 | 0 | 3 |
| 113 | K.FNANWEAFSNHGEFDDTHANSLEAVHDDIHGF.V | 71.0 | 3642.5461 | 911.6433 | -1 | 4 |
| 114 | K.FNANWEAFSNHGEFD.D | 69.6 | 1783.7227 | 892.868 | -1 | 2 |
| 115 | K.FNANWEAFSNHGEFDDTHAN.S | 66.9 | 2321.9363 | 1161.9741 | -1 | 2 |
| 116 | K.FNANWEAFSNHGEFDDTHANSLEAVHD.D | 62.2 | 3073.2861 | 769.3282 | -1 | 4 |
| 117 | K.FNANWEAFSNH.G | 55.6 | 1335.5632 | 668.7883 | -1 | 2 |
| 118 | K.FNANWEAFSNHG.E | 53.9 | 1392.5847 | 697.2982 | -2 | 2 |
| 119 | K.FNANWEAFSNHGEFDDTHANSLEAVHDD.I | 52.6 | 3188.3132 | 798.0862 | 1 | 4 |
| 120 | K.FNANWEAFSNHGEFDDTHV**(sub A)**NSLEAVHDDIHGFVGR.G | 50.5 | 3982.7683 | 996.6991 | 0 | 4 |
| 121 | K.FNANWEAFSNHGEFDDTHANSV**(sub L)**EAVHDDIHA**(sub G)**FVGR.G | 50.1 | 3954.7371 | 660.131 | 1 | 6 |
| 122 | K.FNANWEAF.S | 37.6 | 997.4293 | 499.7212 | -2 | 2 |
| 123 | K.FNANWEAFSN.H | 32.0 | 1198.5043 | 600.2612 | 3 | 2 |
| 124 | N.ANWEAFSNHGEFDDTHANSLEAVHDDIHGFVGR.G | 65.5 | 3693.6257 | 739.7322 | 0 | 5 |
| 125 | N.WEAFSNHGEFDDTHANSLEAVHDDIHGFVGR.G | 71.0 | 3508.5457 | 702.7159 | -1 | 5 |
| 126 | F.SNHGEFDDTHANSLEAVHDDIHGFVGR.G | 103.8 | 2975.3181 | 744.8365 | 0 | 4 |
| 127 | N.HGEFDDTHANSLEAVHDDIHGFVGR.G | 114.9 | 2774.2432 | 694.5679 | 0 | 4 |
| 128 | D.DTHANSLEAVHDDIHGFVGR.G | 94.6 | 2189.0249 | 730.6805 | -2 | 3 |
| 129 | D.THANSLEAVHDDIHGFVGR.G | 93.3 | 2073.998 | 692.3383 | -3 | 3 |
| 130 | N.SLEAVHDDIHGFVGR.G | 87.0 | 1650.8114 | 826.4114 | -2 | 2 |
| 131 | S.LEAVHDDIHGFVGR.G | 49.1 | 1563.7793 | 522.2657 | -3 | 3 |
| 132 | L.EAVHDDIHGFVGR.G | 82.6 | 1450.6953 | 726.3539 | -1 | 2 |
| 133 | D.DIHGFVGR.G | 44.3 | 899.4613 | 450.7374 | -1 | 2 |
| 134 | R.GHMTHALFAAFDPIFWLHHSNVDR.H | 98.0 | 2818.355 | 564.6787 | 1 | 5 |
| 135 | R.GHMTHALFAAFD.P | 56.0 | 1316.5972 | 659.3046 | -2 | 2 |
| 136 | R.GHMTHALF.A | 48.3 | 912.4276 | 913.4343 | -1 | 1 |
| 137 | R.GHMTHALFA.A | 43.0 | 983.4647 | 492.7394 | -1 | 2 |
| 138 | H.MTHALFAAFDPIFWLHHSNVDR.H | 81.6 | 2624.2747 | 525.8625 | 1 | 5 |
| 139 | H.ALFAAFDPIFWLHHSNVDR.H | 86.2 | 2255.1274 | 1128.5703 | -1 | 2 |
| 140 | A.LFAAFDPIFWLHHSNVDR.H | 54.7 | 2184.0903 | 547.0287 | -2 | 4 |
| 141 | L.FAAFDPIFWLHHSNVDR.H | 75.5 | 2071.0063 | 691.3425 | 0 | 3 |
| 142 | F.AAFDPIFWLHHSNVDR.H | 77.1 | 1923.938 | 962.9759 | 0 | 2 |
| 143 | A.AFDPIFWLHHSNVDR.H | 54.4 | 1852.9009 | 464.2321 | -1 | 4 |
| 144 | A.FDPIFWLHHSNVDR.H | 52.8 | 1781.8638 | 446.4728 | -1 | 4 |
| 145 | D.PIFWLHHSNVDR.H | 68.6 | 1519.7684 | 760.891 | -1 | 2 |
| 146 | L.HHSNVDR.H | 16.1 | 863.3998 | 432.7069 | -1 | 2 |
| 147 | R.HLSLWQALYPGVWVTQGPER.E | 83.4 | 2336.2065 | 779.7427 | 0 | 3 |
| 148 | R.HLSLWQALYPGVWVT.Q | 68.3 | 1768.9301 | 885.4719 | -1 | 2 |
| 149 | R.HLSLWQALYPGVWVTQ.G | 68.3 | 1896.9886 | 949.5019 | 0 | 2 |
| 150 | R.HLSLWQA.L | 43.4 | 853.4446 | 854.4506 | -2 | 1 |
| 151 | R.HLSLWQ.A | 30.7 | 782.4075 | 783.4142 | -1 | 1 |
| 152 | R.HLSLWQAL.Y | 18.3 | 966.5287 | 484.273 | 3 | 2 |
| 153 | L.WQALYPGVWVTQGPER.E | 75.2 | 1885.9475 | 943.9799 | -1 | 2 |
| 154 | W.QALYPGVWVTQGPER.E | 82.8 | 1699.8682 | 850.9415 | 0 | 2 |
| 155 | Q.ALYPGVWVTQGPER.E | 82.1 | 1571.8096 | 786.9106 | -2 | 2 |
| 156 | A.LYPGVWVTQGPER.E | 76.3 | 1500.7725 | 751.3933 | 0 | 2 |
| 157 | L.YPGVWVTQGPER.E | 71.2 | 1387.6885 | 694.8508 | -1 | 2 |
| 158 | Y.PGVWVTQGPER.E | 72.1 | 1224.6251 | 613.3197 | 0 | 2 |
| 159 | R.EGSMGFAPGTELNK.D | 70.6 | 1436.6605 | 719.3362 | -2 | 2 |
| 160 | R.EGSMGFAPGTELNKD.S | 69.8 | 1551.6875 | 776.8492 | -2 | 2 |
| 161 | R.EGSMGFAPGTELNKDSALEPFYETEDKPWTSVPLTDTALLNYSYPDFDK.V | 61.7 | 5485.5386 | 1098.1168 | 2 | 5 |
| 162 | R.EGSMGFAPGTELNKDSALEPFYETEDKPWTSVPLTDTALLNY.S | 39.1 | 4633.1733 | 1159.3053 | 4 | 4 |
| 163 | E.GSMGFAPGTELNK.D | 63.9 | 1307.6179 | 654.8148 | -2 | 2 |
| 164 | G.SMGFAPGTELNK.D | 63.8 | 1250.5964 | 626.3041 | -2 | 2 |
| 165 | S.MGFAPGTELNK.D | 66.7 | 1163.5645 | 582.7885 | -2 | 2 |
| 166 | M.GFAPGTELNK.D | 49.7 | 1032.5239 | 517.2684 | -2 | 2 |
| 167 | G.FAPGTELNK.D | 50.4 | 975.5025 | 488.7581 | -1 | 2 |
| 168 | N.KDSALEPFYETEDKPWTSVPLTDTALLNYSYPDFDK.V | 55.0 | 4194.9834 | 1049.7538 | 1 | 4 |
| 169 | K.DSALEPFYETEDKPWTSVPLTDTALLNY.S | 84.0 | 3214.5234 | 1072.5145 | -1 | 3 |
| 170 | K.DSALEPFYETEDKPWT.S | 79.8 | 1926.8523 | 964.4321 | -1 | 2 |
| 171 | K.DSALEPFYETEDKPWTSVPLTDTALL.N | 79.4 | 2937.417 | 980.1472 | 1 | 3 |
| 172 | K.DSALEPFYETEDKPWTSVPLTDTAL.L | 78.1 | 2824.333 | 942.4531 | 2 | 3 |
| 173 | K.DSALEPFYETEDKPWTSVPLTDTA.L | 76.3 | 2711.249 | 904.7565 | -1 | 3 |
| 174 | K.DSALEPFYETEDKPWTSVPLTDTALLNYSYPDFDKVK.G | 73.3 | 4294.0522 | 1074.521 | 1 | 4 |
| 175 | K.DSALEPFYETEDKPWTSVPLTDTALLNYSYPDFDK.V | 72.7 | 4066.8887 | 1017.7263 | -3 | 4 |
| 176 | K.DSALEPFYETEDKPWTSVPLTD.T | 72.4 | 2539.1641 | 847.3951 | 0 | 3 |
| 177 | K.DSALEPFYETEDKPW.T | 72.1 | 1825.8046 | 913.908 | -2 | 2 |
| 178 | K.DSALEPFYETEDK.P | 61.1 | 1542.6725 | 772.3429 | -1 | 2 |
| 179 | K.DSALEPFYETEDKPWTSVPLTDTALLNYSYPDFD.K | 61.1 | 3938.7937 | 1313.9362 | -2 | 3 |
| 180 | K.DSALEPFYETEDKPWTSVPLTDTALLNYSYPD.F | 60.3 | 3676.6985 | 1226.5736 | 0 | 3 |
| 181 | K.DSALEPFYETEDKPWTSVPL.T | 59.0 | 2323.0896 | 1162.5533 | 1 | 2 |
| 182 | K.DSALEPFYETED.K | 47.1 | 1414.5775 | 708.2949 | -2 | 2 |
| 183 | K.DSALEPF.Y | 24.5 | 777.3544 | 778.3608 | -1 | 1 |
| 184 | D.SALEPFYETEDKPWTSVPLTDTALLNYSYPDFDK.V | 73.8 | 3951.8618 | 988.9728 | 0 | 4 |
| 185 | F.YETEDKPWTSVPLTDTALLNYSYPDFDK.V | 74.3 | 3307.5447 | 1103.519 | -3 | 3 |
| 186 | E.DKPWTSVPLTDTALLNYSYPDFDK.V | 78.8 | 2785.3486 | 929.4562 | -1 | 3 |
| 187 | D.KPWTSVPLTDTALLNYSYPDFDK.V | 96.5 | 2670.3218 | 668.5872 | -1 | 4 |
| 188 | K.PWTSVPLTDTALLNYSYPDFDK.V | 88.8 | 2542.2268 | 848.4162 | 0 | 3 |
| 189 | W.TSVPLTDTALLNYSYPDFDK.V | 55.2 | 2259.0947 | 754.0377 | -2 | 3 |
| 190 | T.SVPLTDTALLNYSYPDFDK.V | 72.3 | 2158.0469 | 1080.031 | 0 | 2 |
| 191 | L.TDTALLNYSYPDFDK.V | 67.0 | 1761.8097 | 881.911 | -1 | 2 |
| 192 | D.TALLNYSYPDFDK.V | 78.7 | 1545.7351 | 773.8748 | 0 | 2 |
| 193 | A.LLNYSYPDFDK.V | 74.3 | 1373.6503 | 687.8318 | -1 | 2 |
| 194 | L.LNYSYPDFDK.V | 67.8 | 1260.5662 | 631.2896 | -1 | 2 |
| 195 | L.NYSYPDFDK.V | 51.1 | 1147.4822 | 574.7478 | -1 | 2 |
| 196 | N.YSYPDFDK.V | 56.0 | 1033.4392 | 1034.4457 | -1 | 1 |
| 197 | N.YSYPDFDKVK.G | 50.3 | 1260.6025 | 421.2079 | -1 | 3 |
| 198 | Y.SYPDFDK.V | 42.7 | 870.3759 | 871.3824 | -1 | 1 |
| 199 | Y.PDFDK.V | 22.3 | 620.2806 | 621.2874 | -1 | 1 |
| 200 | V.KGGTPDLVR.D | 37.2 | 941.5294 | 471.773 | 2 | 2 |
| 201 | V.R**(sub K)**GGTPDLVR.D | 33.3 | 969.5355 | 485.7755 | 1 | 2 |
| 202 | K.GGTPDLVR.D | 48.1 | 813.4344 | 814.4399 | -2 | 1 |
| 203 | K.GGTPDLVRD.Y | 40.9 | 928.4614 | 465.2382 | 1 | 2 |
| 204 | G.TPDLVR.D | 18.5 | 699.3915 | 700.3967 | -3 | 1 |
| 205 | R.DYINDHIDR.R | 71.2 | 1159.5258 | 580.7695 | -1 | 2 |
| 206 | R.DYINDHIDRR.Y | 55.2 | 1315.6268 | 658.8185 | -3 | 2 |
| 207 | R.DYINDHID.R | 40.0 | 1003.4247 | 502.7195 | 0 | 2 |
| 208 | D.YINDHIDR.R | 59.6 | 1044.4988 | 523.2564 | -1 | 2 |
| 209 | D.YINDHIDRR.Y | 18.7 | 1200.6 | 401.2066 | -2 | 3 |
| 210 | Y.INDHIDR.R | 34.4 | 881.4355 | 441.7242 | -2 | 2 |
| 211 | K.KSEGGKNPAQDLLSDFK.G | 64.8 | 1832.9268 | 611.9814 | -2 | 3 |
| 212 | K.SEGGKNPAQDLLSDFK.G | 96.7 | 1704.8318 | 569.2841 | -1 | 3 |
| 213 | K.SEGGKNPAL**(sub Q)**DLLSDFK.G | 94.7 | 1689.8573 | 845.9344 | -2 | 2 |
| 214 | G.KNPAQDLLSDFK.G | 23.2 | 1374.7142 | 688.3638 | -1 | 2 |
| 215 | K.NPAQDLLSDFK.G | 86.6 | 1246.6194 | 624.3163 | -1 | 2 |
| 216 | K.NPAL**(sub Q)**DLLSDFK.G | 84.6 | 1231.6448 | 616.8283 | -2 | 2 |
| 217 | K.NPAQDLLSD.F | 41.2 | 971.4559 | 972.4625 | -1 | 1 |
| 218 | K.NPAL**(sub Q)**DLLSD.F | 33.4 | 956.4814 | 479.2481 | 0 | 2 |
| 219 | N.PAQDLLSDFK.G | 78.4 | 1132.5764 | 567.2955 | 0 | 2 |
| 220 | N.PAL**(sub Q)**DLLSDFK.G | 71.5 | 1117.6019 | 559.8078 | -1 | 2 |
| 221 | P.AQDLLSDFK.G | 59.1 | 1035.5237 | 518.7681 | -2 | 2 |
| 222 | P.AL**(sub Q)**DLLSDFK.G | 54.0 | 1020.5491 | 511.2814 | -1 | 2 |
| 223 | K.GVTHDHNEDLK.M | 53.7 | 1263.5844 | 632.8018 | 4 | 2 |
| 224 | K.MFDWTIQASWK.K | 88.3 | 1411.6594 | 706.8365 | -1 | 2 |
| 225 | K.MFDWTIQAS.W | 52.9 | 1097.4852 | 549.7496 | -1 | 2 |
| 226 | K.MFDWTIQ.A | 32.5 | 939.416 | 470.7155 | 0 | 2 |
| 227 | K.KFELDDSFAIIFYFAADGSTNVTK.E | 98.5 | 2698.3167 | 900.4454 | -1 | 3 |
| 228 | K.KFELDDSFAIIFY.F | 61.0 | 1606.7919 | 804.4036 | 1 | 2 |
| 229 | K.KFELDDSF.A | 46.9 | 999.4549 | 500.7343 | -1 | 2 |
| 230 | K.KFELDDSFAIIF.Y | 41.1 | 1443.7285 | 722.8711 | -1 | 2 |
| 231 | K.FELDDSFAIIFY.F | 67.2 | 1478.6969 | 740.3552 | -1 | 2 |
| 232 | F.YFAADGSTNVTK.E | 73.0 | 1272.5986 | 637.3058 | -1 | 2 |
| 233 | Y.FAADGSTNVTK.E | 61.2 | 1109.5353 | 555.7753 | 1 | 2 |
| 234 | F.AADGSTNVTK.E | 49.1 | 962.4669 | 482.2422 | 3 | 2 |
| 235 | K.ENYIGSINIFR.G | 73.6 | 1324.6775 | 663.3457 | -1 | 2 |
| 236 | K.ENYIGSIN.I | 38.6 | 908.424 | 455.2185 | -2 | 2 |
| 237 | N.YIGSINIFR.G | 57.2 | 1081.592 | 541.8026 | -1 | 2 |
| 238 | Y.IGSINIFR.G | 17.5 | 918.5287 | 460.2711 | -1 | 2 |
| 239 | R.TQDNLVQEGFVHLDR.F | 101.3 | 1769.8696 | 885.9407 | -2 | 2 |
| 240 | R.TQDNLVQEGFVHLD.R | 73.4 | 1613.7686 | 807.8899 | -2 | 2 |
| 241 | R.TQDNLVQEGFVH.L | 66.6 | 1385.6575 | 693.8351 | -1 | 2 |
| 242 | R.TQDNLVQEGFVHLDRF.I | 61.8 | 1916.938 | 639.9856 | -2 | 3 |
| 243 | R.TQDNLVQEGF.V | 48.8 | 1149.5302 | 575.772 | -1 | 2 |
| 244 | Q.DNLVQEGFVHLDR.F | 53.7 | 1540.7634 | 514.5947 | -1 | 3 |
| 245 | D.NLVQEGFVHLDR.F | 74.8 | 1425.7365 | 713.8754 | 0 | 2 |
| 246 | N.LVQEGFVHLDR.F | 66.8 | 1311.6935 | 656.8535 | -1 | 2 |
| 247 | V.QEGFVHLDR.F | 37.9 | 1099.541 | 550.7776 | 0 | 2 |
| 248 | R.DLDTFDPQAVHR.Y | 86.3 | 1412.6685 | 707.3412 | 0 | 2 |
| 249 | D.LDTFDPQAVHR.Y | 74.1 | 1297.6415 | 649.8275 | -1 | 2 |
| 250 | L.DTFDPQAVHR.Y | 58.1 | 1184.5574 | 593.2855 | -1 | 2 |
| 251 | D.TFDPQAVHR.Y | 57.7 | 1069.5305 | 535.771 | -3 | 2 |
| 252 | T.FDPQAVHR.Y | 45.4 | 968.4828 | 485.2475 | -2 | 2 |
| 253 | K.VVADDHSVTLK.S | 73.1 | 1182.6244 | 592.319 | -1 | 2 |
| 254 | K.VVADDHSVTLKSLR.I | 43.5 | 1538.8417 | 513.9543 | 0 | 3 |
| 255 | K.VVADDHSVTL.K | 38.0 | 1054.5294 | 528.2722 | 0 | 2 |
| 256 | V.VADDHSVTLK.S | 41.1 | 1083.556 | 542.7853 | 0 | 2 |
| 257 | V.ADDHSVTLK.S | 54.5 | 984.4876 | 493.2507 | -1 | 2 |
| 258 | A.DDHSVTLK.S | 26.1 | 913.4505 | 457.7314 | -2 | 2 |
| 259 | D.DHSVTLK.S | 24.7 | 798.4236 | 400.2172 | -5 | 2 |
| 260 | R.VQGRPLHLPPGVSFPR.L | 44.9 | 1755.9896 | 440.0044 | -1 | 4 |
| 261 | R.PLHLPPGVSFPR.L | 69.2 | 1315.74 | 439.5876 | 1 | 3 |
| 262 | L.HLPPGVSFPR.L | 45.7 | 1105.6033 | 553.808 | -2 | 2 |
| 263 | L.PPGVSFPR.L | 48.1 | 855.4603 | 428.7372 | 0 | 2 |
| 264 | P.PGVSFPR.L | 21.3 | 758.4075 | 759.4134 | -2 | 1 |
| 265 | K.NIPIVNFDDVLDLVTGVVNIGLTAVGAT.A | 81.3 | 2838.5378 | 947.1857 | -1 | 3 |
| 266 | K.NIPIVNFDDVLDLVTGVVNIG.L | 79.0 | 2225.1943 | 1113.604 | 0 | 2 |
| 267 | K.NIPIVNFDDVLDLVTGVVNIGLTAVGATAGVA.I | 78.9 | 3136.7019 | 1046.5756 | 1 | 3 |
| 268 | K.NIPIVNFDDVLDLVTGVVNIGLTAVGATAG.V | 76.9 | 2966.5964 | 989.8721 | -1 | 3 |
| 269 | K.NIPIVNFDDVLDLVTGVVNIGLT.A | 73.8 | 2439.3262 | 1220.6707 | 0 | 2 |
| 270 | K.NIPIVNFDDVLDLVTGVVN.I | 73.1 | 2055.0889 | 686.0361 | -1 | 3 |
| 271 | K.NIPIVNFDDVLDLVTGVVNIGLTAVGATA.G | 72.2 | 2909.575 | 970.8657 | 0 | 3 |
| 272 | K.NIPIVNFDDVLDLVTGVVNIGL.T | 72.1 | 2338.2783 | 1170.1455 | -1 | 2 |
| 273 | K.NIPIVNFDDVLD.L | 68.4 | 1372.6874 | 687.351 | 0 | 2 |
| 274 | K.NIPIVNFDDVLDLVTGVVNIGLTA.V | 67.8 | 2510.3633 | 837.7942 | -1 | 3 |
| 275 | K.NIPIVNFDDVLDLVTGVVNIGLTAVGA.T | 67.3 | 2737.4902 | 913.5027 | -1 | 3 |
| 276 | K.NIPIVNFDDVLDLVTGVVNIGLTAVG.A | 66.9 | 2666.4531 | 889.8245 | -1 | 3 |
| 277 | K.NIPIVNFDDVLDLVTGVVNIGLTAV.G | 62.5 | 2609.4316 | 870.8179 | 0 | 3 |
| 278 | K.NIPIVNFDDVLDLVTGVV.N | 58.5 | 1941.0459 | 971.5297 | -1 | 2 |
| 279 | K.NIPIVNFD.D | 56.2 | 930.4811 | 466.2473 | -1 | 2 |
| 280 | K.NIPIVN.F | 19.1 | 668.3857 | 669.3913 | -3 | 1 |

Table 15a. List of peptides matches found by nano-HPLC ESI-MS/MS measurements for run 5.

| **Reduced sample L-TYR; run 6 (measured by *Proteom Factory*)** | |
| --- | --- |
| Device and operating software used | nanoLC-ESI-MSMS |
| Software used (peak list generating) | Xcalibur 2.2 SP1.48 (*Thermo Scientific*) |
| Acquisition parameters | default |
| **Search Parameters** | |
| Search engine | Mascot Search engine (*Matrix Science*) |
| Enzyme specify | Trypsin |
| Number of miss cleavages permitted | 2 |
| Number of non-specific cleavage | 0 |
| Fixed modifications (including residue specificity) | 0 |
| Variable modifications (including residue specificity) | Methionine oxidation (+15.99).Cysteine propioneamidation (+71.04) |
| Mass tolerance for precursor ions | ±5 ppm |
| Mass tolerance for fragment ions | ±0.6 Dalton |
| Name of database searched (version/date) | NCBInr 110509 |
| Species restriction and justification for searching only a subset of a database | no |
| Number of protein entries in the database actually searched | 13841106 sequences |
| Cut-off score/expectation value for accepting individual MS/MS spectra provided | p ≤ 0.05 |

Table 16a. Experimental settings for nano-HPLC ESI-MS/MS measurements (operated by *Proteome Factory*) and data evaluation for run 5. Sample: Tryptic digest of a gel band (L-TYR. 62 kDa) from a SDS-PAGE under reducing conditions.

| **Protein identification data (run 6)** | | | |
| --- | --- | --- | --- |
| Accession number | Unique peptides detected | Sequence coverage [%] | Score |
| gi\|255687957 polyphenol oxidase [*Agaricus bisporus*] | 61 | 76 | 2604 |
| gi\|6686057 monooxygenase | 4 | 6 | 156 |
| gi\|50289613 hypothetical protein [*Candida glabrata*] | 1 | 1 | 43 |
| gi\|242216727 predicted protein [*Postia placenta*] | 2 | 1 | 43 |
| gi\|119500250 hypothetical protein [*Neosartorya fischeri*] | 2 | 1 | 41 |

Table 17a. List of the five polypeptide sequences exhibiting the highest score for matching to the respective MS-data generated by run 5 (nano-HPLC ESI-MS/MS measurements).

| **Identified peptide list (run 6)** | | | | | | |
| --- | --- | --- | --- | --- | --- | --- |
|  | **Sequence** | **-10lgP** | **m (expected)** | **m/z (observed)** | **ppm** | **z** |
| 1 | M.SLLATVGPTGGVK.N | 43 | 1198.6890 | 600.3518 | -3 | 2 |
| 2 | K.NRLDIVDFVR.D | 12 | 1245.6783 | 623.8465 | -4 | 2 |
| 3 | K.NRLDIVDFVRDEK.F | 19 | 1617.8452 | 540.2890 | -1 | 3 |
| 4 | R.LDIVDFVR.D | 32 | 975.5371 | 488.7758 | -2 | 2 |
| 5 | R.LDIVDFVRDEK.F | 33 | 1347.7025 | 450.2414 | -1 | 3 |
| 6 | R.LDIVDFVRDEKFFTLYVR.A | 38 | 2274.2046 | 759.0755 | 0 | 3 |
| 7 | K.FFTLYVR.A | 25 | 944.5102 | 473.2624 | -2 | 2 |
| 8 | R.ALQAIQDK.D | 44 | 885.4904 | 443.7525 | -2 | 2 |
| 9 | R.ALQAIQDKDQADYSSFFQLSGIHGLPFTPWAKPK.D | 22 | 3803.9220 | 951.9878 | -2 | 4 |
| 10 | K.DQADYSSFFQLSGIHGLPFTPWAKPK.D | 53 | 2936.4466 | 735.1189 | -1 | 4 |
| 11 | R.VYVSIYEQVLQEAAK.G | 100 | 1738.9150 | 870.4648 | 0 | 2 |
| 12 | K.EWVQAAEDLR.Q | 23 | 1215.5876 | 608.8011 | -1 | 2 |
| 13 | R.QPYWDTGFALVPPDEIIK.L | 36 | 2088.0569 | 1045.0357 | 0 | 2 |
| 14 | R.QPYWDTGFALVPPDEIIKLEQVK.I | 28 | 2685.3996 | 896.1405 | -2 | 3 |
| 15 | K.ITNYDGTK.I | 48 | 910.4396 | 456.2271 | 0 | 2 |
| 16 | K.ITNYDGTKITVR.N | 8 | 1379.7389 | 460.9202 | -1 | 3 |
| 17 | K.ITNYDGTKITVRNPILR.Y | 14 | 1973.1026 | 658.7082 | -2 | 3 |
| 18 | K.ITVRNPILR.Y | 25 | 1080.6772 | 361.2330 | 0 | 3 |
| 19 | R.YSFHPIDPSFSGYPNFDTWR.T | 5 | 2432.0930 | 811.7050 | 3 | 3 |
| 20 | R.TTVRNPDADKK.E | 21 | 1243.6550 | 415.5589 | 2 | 3 |
| 21 | R.NPDADKKENIPALIAK.L | 27 | 1735.9487 | 579.6568 | 1 | 3 |
| 22 | K.ENIPALIAK.L | 10 | 967.5704 | 484.7925 | 0 | 2 |
| 23 | K.ENIPALIAKLDLEADSTR.E | 12 | 1968.0510 | 657.0243 | -1 | 3 |
| 24 | K.ENIPALIAKLDLEADSTREK.T | 20 | 2225.1839 | 742.7352 | -3 | 3 |
| 25 | K.LDLEADSTR.E | 35 | 1018.4927 | 1019.5000 | 0 | 1 |
| 26 | K.LDLEADSTREK.T | 30 | 1275.6302 | 426.2173 | 0 | 3 |
| 27 | K.LDLEADSTREKTYNMLK.F | 29 | 2041.9975 | 681.6731 | -1 | 3 |
| 28 | R.EKTYNMLK.F | 45 | 1025.5197 | 513.7671 | -2 | 2 |
| 29 | K.TYNMLK.F | 8 | 768.3835 | 385.1990 | -1 | 2 |
| 30 | K.FNANWEAFSNHGEFDDTHANSLEAVHDDIHGFVGR.G | 21 | 3954.7356 | 989.6912 | 0 | 4 |
| 31 | R.GH**M(+15.99)**THALFAAFDPIFWLHHSNVDR.H | 36 | 2834.3487 | 709.5944 | 0 | 4 |
| 32 | R.HLSLWQALYPGVWVTQGPER.E | 9 | 2336.2032 | 1169.1089 | -1 | 2 |
| 33 | R.EGS **M(+15.99)**GFAPGTELNK.D | 13 | 1452.6558 | 727.3352 | 0 | 2 |
| 34 | K.DSALEPFYETEDKPWTSVPLTDTALLNYSYPDFDKVK.G | 18 | 4294.0438 | 1074.5182 | -2 | 4 |
| 35 | K.VKGGTPDLVR.D | 55 | 1040.5960 | 521.3053 | -2 | 2 |
| 36 | K.VKGGTPDLVRDYINDHIDR.R | 12 | 2182.1084 | 546.5344 | -2 | 4 |
| 37 | K.GGTPDLVR.D | 14 | 813.4342 | 407.7244 | 0 | 2 |
| 38 | K.GGTPDLVRDYINDHIDR.R | 18 | 1954.9472 | 652.6564 | -1 | 3 |
| 39 | K.GGTPDLVRDYINDHIDRR.Y | 14 | 2111.0499 | 704.6906 | 0 | 3 |
| 40 | R.DYINDHIDR.R | 52 | 1159.5230 | 580.7688 | -2 | 2 |
| 41 | R.DYINDHIDRR.Y | 14 | 1315.6273 | 439.5497 | 0 | 3 |
| 42 | K.KSEGGKNPALDLLSDFK.G | 70 | 1817.9514 | 909.9830 | 0 | 2 |
| 43 | K.SEGGKNPALDLLSDFK.G | 28 | 1689.8577 | 564.2932 | 0 | 3 |
| 44 | K.SEGGKNPALDLLSDFKGVTHDHNEDLK.M | 16 | 2935.4261 | 734.8638 | -2 | 4 |
| 45 | K.NPALDLLSDFK.G | 25 | 1231.6454 | 616.8300 | 0 | 2 |
| 46 | K.GVTHDHNEDLK.M | 23 | 1263.5845 | 422.2021 | 0 | 3 |
| 47 | K.GVTHDHNEDLK **M(+15.99)**FDWTIQASWK.K | 9 | 2673.2275 | 669.3142 | 0 | 4 |
| 48 | K.MFDWTIQASWK.K | 31 | 1427.6575 | 714.8360 | 2 | 2 |
| 49 | K.ENYIGSINIFR.G | 38 | 1324.6779 | 663.3462 | 0 | 2 |
| 50 | R.GTTPTN**C(+71.04)**AN **C(+71.04)**R.T | 41 | 1278.5462 | 640.2804 | 1 | 2 |
| 51 | R.TQDNLVQEGFVHLDR.F | 80 | 1769.8691 | 590.9636 | 0 | 3 |
| 52 | R.TQDNLVQEGFVHLDRFIAR.D | 22 | 2257.1575 | 565.2966 | -1 | 4 |
| 53 | R.FIARDLDTFDPQAVHR.Y | 39 | 1899.9555 | 475.9962 | -2 | 4 |
| 54 | R.DLDTFDPQAVHR.Y | 26 | 1412.6692 | 471.8970 | 0 | 3 |
| 55 | K.KLSYKVVADDHSVTLK.S | 49 | 1801.9918 | 601.6712 | -1 | 3 |
| 56 | K.LSYKVVADDHSVTLK.S | 37 | 1673.8968 | 419.4815 | -1 | 4 |
| 57 | K.VVADDHSVTLK.S | 30 | 1182.6242 | 395.2153 | 0 | 3 |
| 58 | R.IRVQGRPLHLPPGVSFPR.L | 13 | 2025.1707 | 406.0414 | -2 | 5 |
| 59 | R.VQGRPLHLPPGVSFPR.L | 13 | 1755.9876 | 586.3365 | -1 | 3 |
| 60 | R.VQGRPLHLPPGVSFPRLDK.N | 8 | 2112.1951 | 529.0561 | 0 | 4 |
| 61 | R.PLHLPPGVSFPR.L | 12 | 1315.7391 | 658.8768 | -1 | 2 |

Table 18a. List of peptides matches found by nano-HPLC ESI-MS/MS measurements for run 5.

| **Reduced sample light bands (PPO4 fragments, *ab*Lectin Q00022) ; run 7 (measured by *Proteom Factory*)** | |
| --- | --- |
| Device and operating software used | nanoLC-ESI-MSMS |
| Software used (peak list generating) | Xcalibur 2.2 SP1.48 (*Thermo Scientific*) |
| Acquisition parameters | default |
| **Search Parameters** | |
| Search engine | Mascot Search engine (*Matrix Science*) |
| Enzyme specify | Trypsin |
| Number of miss cleavages permitted | 2 |
| Number of non-specific cleavage | 0 |
| Fixed modifications (including residue specificity) | 0 |
| Variable modifications (including residue specificity) | Methionine oxidation (+15.99), Carbamidomethyl (+57.02), Asparagine deamidated (+0.98), Cysteine propioneamidation (+71.04). |
| Mass tolerance for precursor ions | ±3 ppm |
| Mass tolerance for fragment ions | ±0.6 Dalton |
| Name of database searched (version/date) | NCBInr 2013 |
| Species restriction and justification for searching only a subset of a database | no |
| Number of protein entries in the database actually searched | 34305433 sequences |
| Cut-off score/expectation value for accepting individual MS/MS spectra provided | p ≤ 0.05 |

Table 19a. Experimental settings for nano-HPLC ESI-MS/MS measurements (operated by Proteome Factory) and data evaluation for run 7. Sample: Tryptic digest of a gel bands (A-TYR. ~15 kDa) from a SDS-PAGE under reducing conditions.

| **Protein identification data (run 7)** | | | |
| --- | --- | --- | --- |
| Accession number | Unique peptides detected | Sequence coverage [%] | Score |
| gi\|384950716 polyphenol oxidase 4 [*Agaricus bisporus*] | 23 | 30 | 1117 |
| gi\|61679569 Chain A, Common Edible Mushroom (Agaricus Bisporus) Lectin | 12 | 73 | 988 |
| gi\|171684547 hypothetical protein [Podospora anserina S mat+] | 1 | 3 | 69 |
| gi\|6686057 Polyphenol oxidase 2 [Agaricus bisporus] | 2 | 4 | 62 |
| gi\|154275012 predicted protein [Ajellomyces capsulatus NAm1] | 1 | 2 | 53 |

Table 20a. List of the five polypeptide sequences exhibiting the highest score for matching to the respective MS-data generated by run 7(nano-HPLC ESI-MS/MS measurements).

| **Identified peptide list (run 7)** | | | | | | |
| --- | --- | --- | --- | --- | --- | --- |
|  | **Sequence** | **-10lgP** | **m (expected)** | **m/z (observed)** | **ppm** | **z** |
|  | gi\|384950716 peptides: |  |  |  |  |  |
| 1 | K.FFTLYVR.A | 43 | 944.5131 | 473.2638 | 1 | 2 |
| 2 | K.LDLEADSTR.E | 57 | 1018.4928 | 510.2537 | 0 | 2 |
| 3 | R.DYINDHIDR.R | 51 | 1159.5225 | 580.7685 | -3 | 2 |
| 4 | K.VVADDHSVTLK.S | 82 | 1182.6213 | 592.3179 | -3 | 2 |
| 5 | K.NRLDIVDFVR.D | 28 | 1245.6823 | 416.2347 | -1 | 3 |
| 6 | K.LDLEADSTREK.T | 33 | 1275.6284 | 426.2167 | -2 | 3 |
| 7 | R.DYINDHIDRR.Y | 55 | 1315.6266 | 658.8206 | 0 | 2 |
| 8 | R.PLHLPPGVSFPR.L | 50 | 1315.7402 | 439.5873 | 0 | 3 |
| 9 | R.DYI**N(+0.98)**DHIDRR.Y | -24 | 1316.6119 | 439.8779 | 1 | 3 |
| 10 | R.DEKFFTLYVR.A | 20 | 1316.6773 | 439.8997 | 1 | 3 |
| 11 | K.ENYIGSINIFR.G | 56 | 1324.6771 | 663.3458 | 0 | 2 |
| 12 | R.LDIVDFVRDEK.F | 38 | 1347.7053 | 674.8599 | 1 | 2 |
| 13 | R.DLDTFDPQAVHR.Y | 59 | 1412.6707 | 707.3426 | 2 | 2 |
| 14 | R.EGSMGFAPGTELNK.D | 34 | 1436.6618 | 719.3382 | 1 | 2 |
| 15 | K.NRLDIVDFVRDEK.F | 47 | 1617.8449 | 809.9297 | -2 | 2 |
| 16 | R.VYVSIYEQVLQEAAK.G | 103 | 1738.9158 | 870.4652 | 1 | 2 |
| 17 | R.VQGRPLHLPPGVSFPR.L | 27 | 1755.9898 | 586.3372 | 0 | 3 |
| 18 | R.TQDNLVQEGFVHLDR.F | 65 | 1769.8695 | 590.9638 | 0 | 3 |
| 19 | K.GGTPDLVRDYINDHIDR.R | 49 | 1954.9497 | 652.6572 | 0 | 3 |
| 20 | R.IRVQGRPLHLPPGVSFPR.L | 26 | 2025.1756 | 507.3012 | 0 | 4 |
| 21 | K.GGTPDLVRDYINDHIDRR.Y | 36 | 2111.0506 | 704.6908 | 0 | 3 |
| 22 | K.GGTPDLVRDYI**N(+0.98)**DHIDRR.Y | -18 | 2112.0329 | 705.0182 | -1 | 3 |
| 23 | R.HLSLWQALYPGVWVTQGPER.E | 102 | 2336.2029 | 1169.1087 | -2 | 2 |
| 24 | R.GHMTHALFAAFDPIFWLHHSNVDR.H | -29 | 2818.3594 | 705.5971 | 2 | 4 |
| 25 | R.GH**M(+15.99)**THALFAAFDPIFWLHHSNVDR.H | 56 | 2834.3507 | 567.8774 | 0 | 5 |
|  | gi\|61679569 peptides: |  |  |  |  |  |
| 1 | K.ANLIIG.- | 45 | 599.3648 | 600.372 | 1 | 1 |
| 2 | TYTISIR.V | 52 | 852.4702 | 427.2424 | 0 | 2 |
| 3 | K.GFFRPVER.T | 24 | 1006.5339 | 504.2742 | -1 | 2 |
| 4 | K.YANGGTWDEVR.G | -77 | 1266.561 | 634.2878 | -1 | 2 |
| 5 | K.YA**N(+0.98)**GGTWDEVR.G | 93 | 1267.5431 | 634.7788 | -3 | 2 |
| 6 | R.ENQLTSYNVANAK.G | 107 | 1450.7078 | 726.3612 | 2 | 2 |
| 7 | R.ENQLTSYNVA**N(+0.98)**AK.G | -93 | 1451.6876 | 726.8511 | -1 | 2 |
| 8 | R.FAIEYTVTEGDNLK.A | 119 | 1598.7836 | 800.3991 | 0 | 2 |
| 9 | R.GEYVLTMGGSGTSGSLR.F | -125 | 1670.795 | 836.4048 | 1 | 2 |
| 10 | R.GEYVLT**M(+15.99)**GGSGTSGSLR.F | 127 | 1686.7845 | 844.3995 | -2 | 2 |
| 11 | R.RFAIEYTVTEGDNLK.A | 79 | 1754.8808 | 878.4477 | -2 | 2 |
| 12 | R.DQARENQLTSYNVANAK.G | 59 | 1920.9255 | 641.3158 | -2 | 3 |
| 13 | R.FVSSDTDESFVATFGVHNYK.R | 86 | 2249.0341 | 1125.5243 | 3 | 2 |
| 14 | R.FVSSDTDESFVATFGVHNYK.R | 91 | 2405.1322 | 1203.5734 | 1 | 2 |
| 15 | R.FVSSDTDESFVATFGVH**N(+0.98)**YKR.W | -14 | 2406.1145 | 803.0454 | 1 | 3 |
| 16 | K.YANGGTWDEVRGEYVLTMGGSGTSGSLR.F | -60 | 2919.3468 | 974.1229 | 0 | 3 |
| 17 | K.YA**N(+0.98)**GGTWDEVRGEYVLTMGGSGTSGSLR.F | -84 | 2920.3375 | 974.4531 | 3 | 3 |
| 18 | K.YANGGTWDEVRGEYVLT**M(+15.99)**GGSGTSGSLR.F | -92 | 2935.3392 | 979.4537 | 0 | 3 |
| 19 | K.YA**N(+0.98)**GGTWDEVRGEYVLT**M(+15.99)**GGSGTSGSLR.F | 93 | 2936.3302 | 979.784 | 2 | 3 |

Table 21a. List of peptides matches found by nano-HPLC ESI-MS/MS measurements for run 7.

1. **MS-data of intact protein (non-denaturating)**


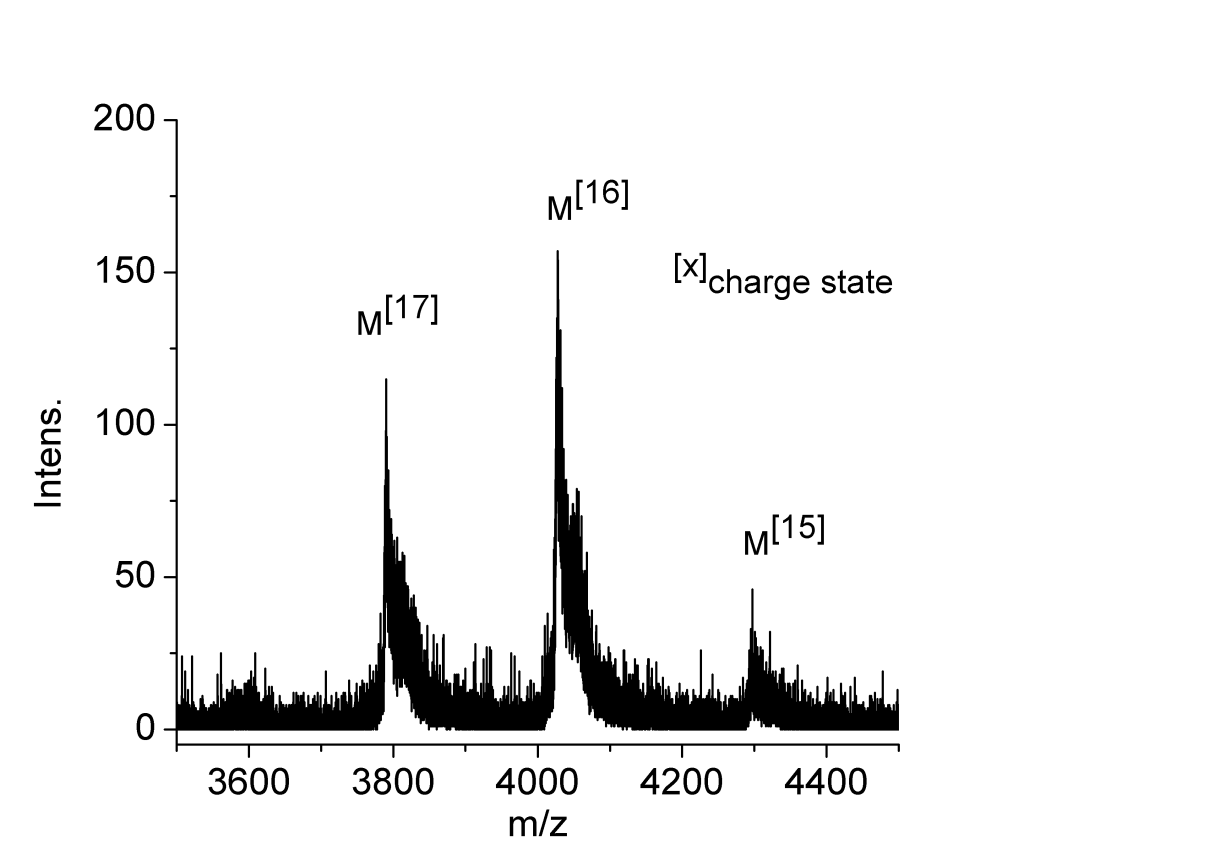


Figure 1b. ESI-QTOF mass spectra of native L-TYR (PPO4) eventuated from a non-acidic treatment (5 mM ammonium acetate buffer pH 5.5). Peaks of the charge state [15] to [17] are shown. M = 64.415.6 Da (STD less than 7.2 Da). Mass differences between denaturated L-TYR (25 % (v/v) ACN. 0.05 % (v/v) formic acid. 5 mM ammonium acetate buffer pH 5.5) and native L-TYR is approximately 165 Da. fitting properly to the calculated weight of the type-3 copper center in its *oxy*-state (2xCu + O_2_^2-^. 159.1 Da).

1. **Basic calculated data (*i*P, Mr) of PPO1-6**

|  |  | PPO1 | PPO2 | PPO3 | PPO4* | PPO5 | PPO6 |
| --- | --- | --- | --- | --- | --- | --- | --- |
| Residues | L-TYR | 1-568 | 1-556 | 1-576 | 2-565 | 1-576 | 1-640 |
|  | A-TYR | 1-385 | 1-380 | 2-392 | 2-383 | 1-404 | 1-422 |
| M_r_ [Da] | L-TYR | 63.897.6 | 63.926.9 | 66.266.5 | 64.247.3 | 66.165.4 | 73.881.7 |
|  | A-TYR | 43.474.4 | 43.819.4 | 45.270.8 | 43.673.3 | 46.843.5 | 48.519.3 |
| *p*I | L-TYR | 6.58 | 5.92 | 5.14 | 5.77 | 5.45 | 6.15 |
|  | A-TYR | 6.35 | 5.97 | 5.15 | 5.58 | 5.27 | 7.19 |

Table 19c. Basic calculated data for A-TYR and L-TYR of PPO1 to 6 (ProtParam. Expasy.org) Start / end of amino acid residue. Molecular weight [Da]. Isoelectric point. *PPO4 data includes the here described alterations.
